# Beyond Binary: Cardiac Patterning as Probabilistic Fate Restriction

**DOI:** 10.1002/bies.70163

**Published:** 2026-08-19

**Authors:** Kenzo Ivanovitch

**Affiliations:** ^1^ Developmental Biology of Birth Defects, Institute of Child Health University College London London UK

**Keywords:** cardiac progenitors, cell fate determination, embryonic heart, epiblast, fate mapping, gastrulation, lineage tracing, live cell imaging, mesoderm, primitive streak

## Abstract

The mammalian heart is classically described as arising from two cardiac lineages, first and second. Yet, recent lineage tracing of Mesp1 mesoderm and live imaging show that cardiac progenitors are already partly biased toward specific heart regions during gastrulation, despite extensive cell mixing. We propose that cardiac fate is probabilistic, analogous to stochastic fate strategies in the retina. Epiblast cells carry probability distributions over fates that resolve during gastrulation into specific regional allocations. Reanalyzing a retrospective clonal dataset, we find that the clones do not single out one fixed lineage tree. Instead, the data permit a family of 361 distinct restriction topologies, none dominant, and only 40 of these are binary. Among the binary trees, the best‐supported recover both where progenitors sit along the proximal–distal axis of the streak and the order in which they leave it, as recent prospective lineage tracing shows. Rather than replacing the first/second lineage concept, this framework shifts the focus from fixed lineage identity to fate probabilities. Testing this idea will require prospective live imaging from epiblast through heart tube formation.

## Graphical abstract

Are cardiac progenitors committed to fixed lineages, or do they carry probability distributions over heart regions in the epiblast that resolve during gastrulation? Reanalyzing mouse retrospective clonal data, we find no single lineage tree, instead 361 distinct restriction topologies, consistent with a probabilistic model where epiblast position set fate probabilities.

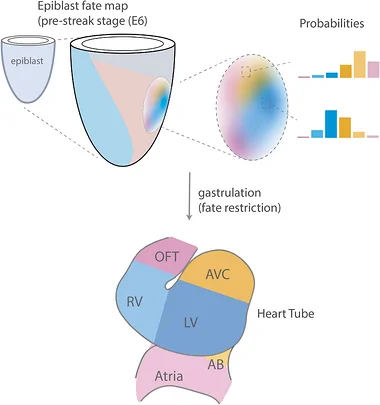

## 1 Introduction: From Binary Trees to Probabilistic Landscapes

How do embryonic organs achieve reproducible patterning from initially similar progenitor cells? The classical view explains this with a series of binary branch points. At each step a progenitor commits to one lineage. Progenitor pools split into distinct lineages, each generating a fixed set of cell types. In the mammalian heart, this view has been formalized as the “first and second cardiac lineages” concept, in which two major lineages diverge early from a common precursor and contribute, with partial overlap, to different regions along the arterial–venous axis of the heart [1].

Studies of other tissues point to a different picture. In the vertebrate retina, precise tissue‐level cell‐type proportions arise not from deterministic lineage trees but from progenitors that behave stochastically. Individual clones differ in size and cell‐type composition, yet the population maintains stable ratios [3]. Each clone is a sample from this stochastic process, so clone‐to‐clone variability reflects probabilistic fate choice. Developmental precision at the population level therefore does not require determinism at the clonal level. Whether the same could be true for the developing heart is the question we examine here. We propose that epiblast cells carry probability distributions over fates, and that during gastrulation these distributions resolve into specific regional allocations.

## 2 Cardiac Fate Biases Are Detectable From Gastrulation

For decades, clonal analysis has shown which descendants of a single cell go where in the embryo [4, 5]. Such studies suggested that before and during gastrulation, epiblast cells mix extensively and remain multipotent, with clonal descendants not confined to a single germ layer [6, 7]. Mesp1‐based clonal analysis in the mouse changed this picture [8, 9]. Mesp1 expression turns on in nascent mesoderm shortly after cells ingress through the primitive streak, well before heart‐tube formation [10]. Yet, Mesp1‐derived clones are already highly restricted, confined to small regions of the heart. Cardiac fate bias can therefore emerge within mesoderm as cells begin to migrate, long before the heart tube becomes segmented, and despite considerable dispersal of sister cells during migration [11].

Using light‐sheet imaging of the Mesp1 lineage, Dominguez and colleagues showed that Mesp1^+^ nascent mesoderm cells mix extensively, with neighbor relationships not preserved. Cells settle into position only as the cardiac crescent emerges [11]. Clonal analysis reports where descendants end up. Live imaging of the mesoderm reports how cells move past one another. A cell can move through changing neighborhoods and still contribute descendants to a confined territory, provided its trajectory is directed toward that territory.

In our recent work, we used light‐sheet imaging to track single mesodermal cells from gastrulation to heart‐tube stage continuously [12]. This allowed us to link each cell's initial position and migratory path to its final descendant locations in the heart tube. Migration paths were organized with respect to fate. We identified uni‐fated progenitors whose daughters contributed only to the Left ventricle/Atrio‐ventricular canal (LV/AVC) or only to inflow/atrial territories, and bipotent progenitors whose daughters contributed to additional mesoderm fates. The uni‐fated progenitors followed more coherent trajectories within the otherwise disordered migratory pattern. Trajectory data alone, however, cannot tell us whether these patterns reflect pre‐existing differences between progenitors or the shared environment of sisters that stay close.

Across 91 complete lineage trees, not one contained both LV/AVC and inflow/atrial cells at the analyzed heart‐tube stage [12]. Tracked progenitors generated small clones (maximum 20 cells per clone, with up to 13 LV/AVC and 8 inflow/atrial descendants) with limited dispersion within the heart tube, consistent with Mesp1 clonal results [9, 13]. Higher‐throughput tracking with live reporters that resolve the boundary between the LV/AVC and inflow/Atria will be needed to rule out rare mixing. This restricted distribution at heart‐tube stage does not by itself imply fate determination in the mesoderm. The heart tube continues to grow, reshape and loop after the stages we imaged, and clones may spread or rearrange further during later morphogenesis [14]. The regional pattern we observe is therefore a snapshot at heart‐tube stage rather than a necessarily fixed assignment imposed in the mesoderm.

**FIGURE 1:**
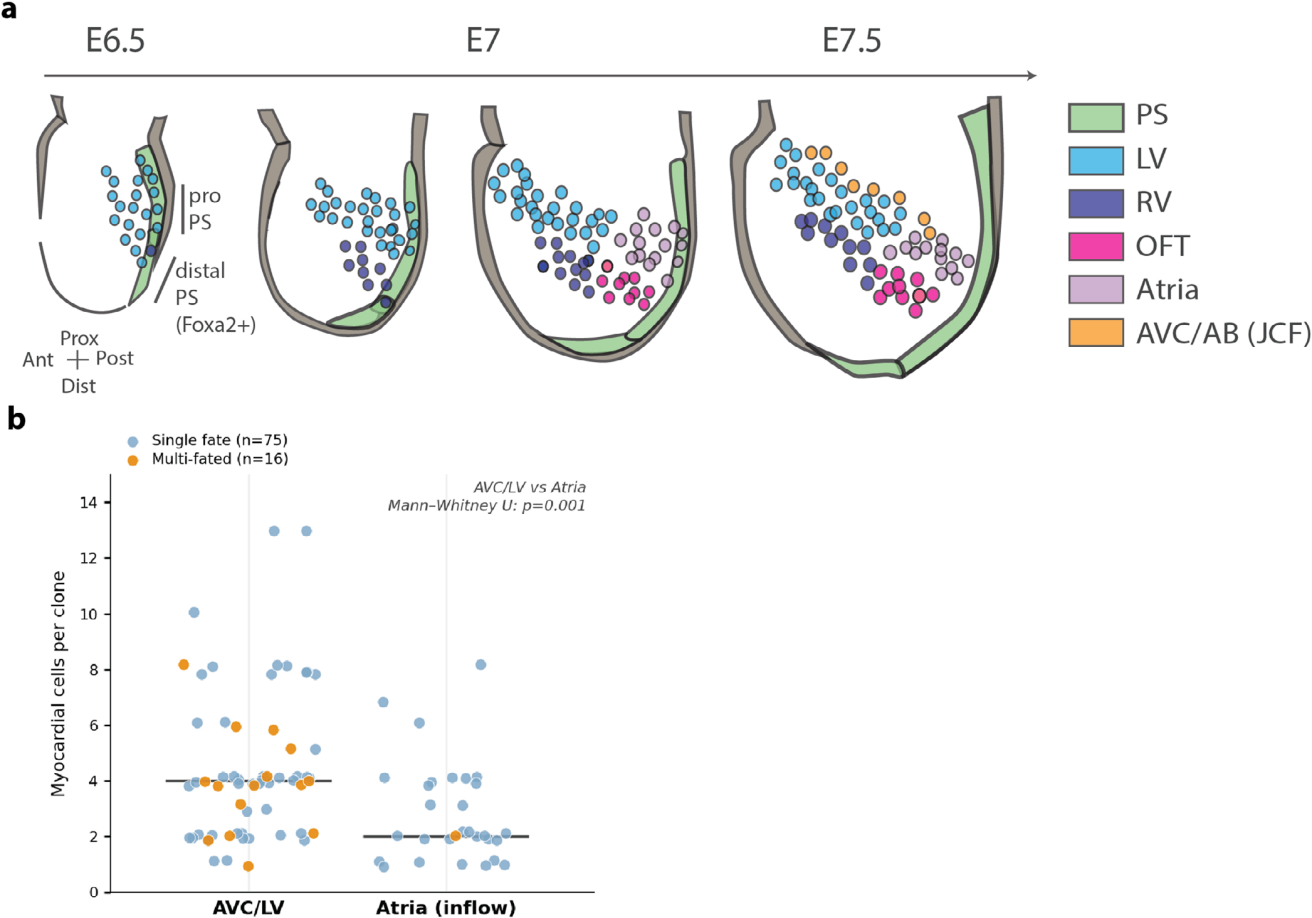
(a) Spatiotemporal model of cardiac progenitor emergence from the primitive streak, deduced from T/Bra and Foxa2 lineage tracing combined with light‐sheet live imaging of nascent mesoderm (adapted from Ivanovitch et al.; Abukar et al.) [12, 15]. Proximal posterior streak cells contribute to LV (earliest) and atria; distal streak cells contribute to LV, RV, and OFT. The origin of the JCF remains uncertain but may derive from the early Hand1+ proximal mesoderm [18]. (b) Myocardial cell counts per clone from live imaging (Abukar et al.), grouped by cardiac identity (AVC/LV or Atria/inflow). *Y*‐axis counts only myocardial descendants. Dots are colored by clone composition: single‐fate (only myocardial cells, blue, *n* = 75) or multi‐fated (at least one non‐myocardial cell type, orange, *n* = 16). Horizontal bars indicate per‐group medians. AVC/LV clones are significantly larger than Atria clones (Mann–Whitney U, two‐sided, *p* = 0.001). AB, atrial body; AVC, atrioventricular canal; JCF, juxtacardiac field; LV, left ventricle; OFT, outflow tract; PS, primitive streak; RV, right ventricle.

Beyond live imaging, *T/Bra* and *Foxa2* temporal lineage tracing combined with scRNA‐seq showed that cardiac progenitors leave the primitive streak in sequential, molecularly distinct cohorts. Each cohort's ingression position predicts its cardiac destination [15]. The spatiotemporal pattern is shown in Figure 1a. LV‐biased cells emerge first from the proximal streak, while right ventricle (RV)‐biased cells emerge from more distal regions. outflow tract (OFT) and atrial progenitors ingress later, at similar stages but from different sites. OFT ingress from the distal streak and atria from the proximal streak [15, 16, 17]. These molecularly distinct, positionally ordered cohorts therefore point to a regional fate bias already present before the heart tube forms.

Single‐cell studies further resolve this partitioning into distinct molecular subpopulations, including early and late Mesp1^+^ progenitors [13, 18, 19]. The nascent mesoderm is also partitioned along its proximal–distal axis into MSX1^+^ and FOXC2^+^ compartments, with the Smarcd3‐F6 enhancer–defined population marking the earliest cardiac progenitors within the proximal embryonic portion. Later, at cardiac crescent stage, the juxtacardiac field (JCF), a Mab21l2^+^/Hand1^+^ population at the embryonic–extraembryonic boundary, is established. These subpopulations are usually interpreted within the established first and second heart field model. The model has been progressively extended to accommodate each new population. How the molecular subpopulations relate to the two heart fields remains an active question, reviewed in detail elsewhere [20].

By the mesoderm stage, thus, cardiac progenitors are already biased toward specific fates, with distinct molecular signatures prefiguring their anatomical destinations, while retaining broader developmental competence as transplantation studies have shown [7]. The heart assembles not from a single multipotent pool, but from several mesodermal subpopulations that are already distinct during gastrulation.

## 3 Revisiting Classic Clonal Data

If cardiac progenitors are already biased at the mesoderm stage, what would this look like at the earlier epiblast stage, before or at the onset of gastrulation? The 2004 retrospective clonal analysis by Meilhac et al. provides a dataset to explore this question [1].

Their study reported a wide range of clone sizes and interpreted this as reflecting different induction timing. Early recombination generated large, widely dispersed clones across multiple heart regions, whereas later recombination generated smaller, more regionally confined clones.

The large clones, labelled at earlier epiblast stages do not simply contribute to all cardiac compartments [1]. Each occupies specific combinations of regions. Some contribute to left ventricle and atria but not outflow tract; others to outflow tract and right‐sided structures but not left ventricle. Even these early clones include some regions and exclude others, suggesting regional bias is already present in the epiblast.

The classic interpretation of these patterns is that two major myocardial lineages, “first” and “second,” diverge early from a common precursor, matching the first and second heart field concept from prospective genetic tracing [21, 22, 23, 24, 25]. But a two‐lineage model may not be the only explanation. The same clonal patterns could arise from a pool of epiblast progenitors with different regional biases akin to the probabilistic strategy seen in the retina.

We therefore re‐examined the assumption that early induced clones reflect a single multipotent pool that bifurcates in two. Our aim was not to replace the first/second lineages and heart fields framework, which remains a useful descriptive and experimental model [20, 26]. We wanted to address whether the clonal data are compatible with a framework in which progenitors differ in their probabilities of contributing to each region, and these biases are narrowed in the mesoderm. We quantified regional co‐occurrence, clone size and regional pattern, and used these as constraints to enumerate every lineage topology compatible with the dataset, rather than fitting a single tree. Future experiments will be needed to discriminate among the candidates lineage trees.

**BOX 1 | Technical Caveats of the LaacZ Clonal Analysis**

*Clone size as a proxy for labelling time*: Clone size is treated throughout as a proxy for when the progenitor was labelled, on the assumption of approximately exponential growth at a roughly constant division rate (∼7 h/division) [1]. Variable proliferation among co‐induced clones introduces noise. Clone‐to‐clone size differences reflect both labelling time and stochastic variation in expansion rate. We therefore use size only in coarse bins (≤30 vs. >30 cells, with sensitivity tested across cutoffs from 20 to 40).

*Double recombination (clumping)*: Two independently labelled cells in the same embryo could in principle be counted as one clone. Meilhac et al. estimated the recombination rate at 3.6 × 10^−^^6^ per cell per division by Luria–Delbrück fluctuation analysis, predicting only ∼8 double‐recombination events across the full dataset [1]. Clumping is therefore statistically rare. Where it occurs, it would tend to inflate the number of regions covered by a clone. It could therefore inflate positive co‐occurrence z‐scores and attenuate the negative OFT–LV coupling we observe.

*Asterisked 1–2 cell contributions*: The original dataset flagged 1–2 cell contributions to a region with asterisks, on the grounds that Luria–Delbrück statistics make these more likely to reflect independent secondary recombination than true clonal descent [1]. Following Meilhac et al., we set asterisked contributions to zero in the binary clone‐by‐region matrix used for co‐occurrence analysis. Had we included asterisks, more clones would appear multi‐regional. In the potency‐graph analysis, this means we may under‐represent the number of clones contributing to many regions.

*Counting dropout*: Individual labelled cells can be missed. Regional presence/absence is robust to dropout of small numbers of cells, since a region only needs one identified descendant to register as positive. Dropout has more effect on clone size, reinforcing that size is used here as an approximation rather than a precise measure of time.

## 4 Clone Size and Regional Occupancy

We first asked whether clone size correlates with the number of cardiac regions a clone occupies. A strong positive correlation would be consistent with a hierarchical model in which a multipotent progenitor sits at the top and regional restriction proceeds gradually with development. To distinguish clones induced at epiblast versus mesodermal stages, we empirically grouped clones into two size bins: small (1–30 cells) and large (≥31 cells). The 30‐cell cutoff was informed by our live‐imaging dataset, where E6.5–E7 mesodermal progenitors generated at most 13 LV/AVC and 8 atrial/inflow heart‐tube descendants (Figure 1b) [12]. One further round of division between heart‐tube and E8.5 looped‐heart stages places the upper bound for mesoderm‐stage induction at ∼25–30 cells. This binning relies on clone size as a proxy for labelling time. Because proliferation rates vary, this proxy is approximate rather than exact, and retrospective clonal analysis carries further limitations (Box 1).

We excluded the largest clone (142 cells, contributing to all six regions), which likely arose from a very early recombination event and would not be informative about mutual exclusion between regions, leaving 93 clones. Of these, nearly half (46/93, 49%) are confined to a single region, almost all of them in the small bin (45/46, 98%).

**FIGURE 2:**
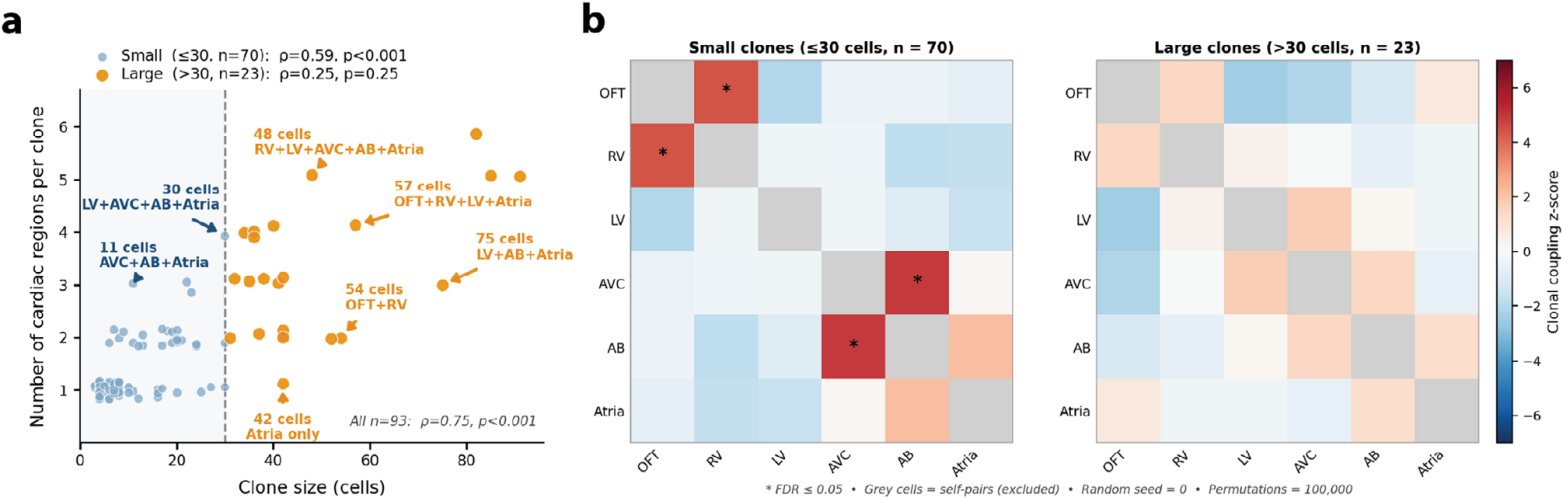
Clone size, regional breadth, and pairwise co‐occurrence. (a) Clone size vs. number of cardiac regions occupied (OFT, RV, LV, AVC, AB, Atria) for *n* = 93 E8.5 clones from Meilhac et al. (Table S1). Clones are split at 30 cells into Small (*n* = 70; blue) and Large (*n* = 23; orange). Spearman correlations per bin and overall are shown in the legend. Labelled examples in the Large bin highlight clones of comparable size with widely different regional compositions. (b) Pairwise clonal‐coupling z‐scores for all 15 region pairs, computed separately for Small (left) and Large (right) clones. Z‐scores quantify how often two regions co‐occur within a clone relative to a null that preserves the number of regions per clone and the number of clones per region (100 000 permutations, seed = 0). Warm colors, positive coupling; cool colors, negative coupling. Asterisks mark pairs significant after FDR correction (FDR ≤ 0.05). Grey diagonal cells are self‐pairs (excluded). AB, atrial body; AVC, atrioventricular canal; LV, left ventricle; OFT, outflow tract; RV, right ventricle.

Across these 93 clones, size and the number of cardiac regions occupied are strongly correlated overall (*ρ* = 0.75, *p* = 4.3 × 10^−^^1^ ^8^; Figure 2a and Table S1). But this correlation is carried by the small clones. Within the small bin it is weaker (*ρ* = 0.59, *p* = 9.5 × 10^−^^8^), and within the large bin, size and region count are independent (*ρ* = 0.25, *p* = 0.25).

Among the larger clones, size no longer predicts regional breadth. Among five 42‐cell clones, one is confined to the atria and another spans AVC, LV and RV. A 54‐cell clone is restricted to OFT and RV, while a 57‐cell clone spans OFT, RV, LV, and Atria. A 48‐cell clone contributes to five regions, whereas a substantially larger 75‐cell clone covers only three (LV, Atria body (AB), and Atria). Clones of comparable size therefore have very different regional compositions, which is hard to reconcile with a strict lineage tree undergoing progressive regional restriction over time.

## 5 Regional Co‐Occurrence

We next quantified regional co‐occurrence within clones to ask whether specific cardiac regions tend to appear together or to be mutually excluded more often than expected by chance. For each region pair, we computed a clonal coupling z‐score, a measure of how often two regions co‐occur in clones, relative to a null model that preserves how many regions each clone occupies and how many clones each region appears in (see Supporting Information).

Following Meilhac et al., asterisk‐flagged 1–2 cell contributions were set to zero in the binary clone‐by‐region matrix. This is a conservative choice. Including them would make clones appear more multi‐regional, so it can under‐represent the most multipotent states (see Box 1).

In large clones (31–92 cells, *n* = 23), no regional pairs reached FDR‐corrected significance, reflecting limited statistical power in this smaller subgroup (Figure 2b). The strongest trend was a modest negative coupling for OFT–LV and OFT–AVC (*z* = −2.44 and −2.16; FDR = 0.22 and 0.23, respectively, Figure S1, Tables 1 and S2), with OFT+LV and OFT+AVC dual clones each appearing in 2 of 23 large clones. With no pair significant, we cannot conclude from this sample that OFT and LV are mutually excluded clonally.

**TABLE 1:** Robustness of large‐clone co‐occurrence to the small/large size threshold. Clonal‐coupling z‐scores for all 15 region pairs in the Large‐clone bin, computed at five alternative size thresholds (> 20, > 25, > 30, > 35, > 40 cells; columns). Cells with FDR ≤ 0.05 are marked with an asterisk. The 30‐cell column is the threshold used in the main text. Z‐scores and FDR are computed as in Figure 2b. Across all five cutoffs, only OFT–LV at the > 20‐cell threshold reaches significance (*z* = −3.23, FDR = 0.018).

Region pair	>20 cells	>25 cells	>30 cells	>35 cells	>40 cells
OFT‐RV	1.18	1.80	1.42	0.67	0.80
AVC‐AB	2.03	1.49	1.46	0.85	0.68
OFT‐LV	−3.23*	−2.64	−2.44	−2.33	−1.27
AB‐Atria	1.99	1.74	1.23	1.75	1.48
OFT‐AVC	−1.56	−1.92	−2.16	−1.68	−1.78
OFT‐AB	−0.32	−1.27	−1.10	−0.76	−1.12
OFT‐Atria	0.33	0.80	0.74	0.38	−0.72
RV‐LV	0.81	−0.08	0.38	0.70	0.17
RV‐AVC	0.02	0.27	0.01	0.93	0.81
RV‐AB	−1.48	−0.97	−0.64	−0.11	−0.14
RV‐Atria	−0.60	−0.03	−0.29	−0.66	−0.68
LV‐AVC	1.61	1.55	1.73	1.34	1.70
LV‐AB	−0.51	−0.24	0.27	0.22	1.06
LV‐Atria	−0.76	−0.89	−0.30	−0.96	−0.18
AVC‐Atria	0.27	−0.02	−0.56	−0.55	−1.23

In small clones (1–30 cells, *n* = 70), two pairs showed significant positive co‐occurrence after FDR correction. These are OFT with RV, and AVC with AB (OFT–RV: FDR = 8.7 × 10^−^^5^; AVC–AB: FDR = 9 × 10^−^^6^; Figure 2b and Table 2). Positive OFT–RV and AVC–AB co‐occurrence in the small bin remained FDR‐significant at every cutoff tested (Table 2; OFT–RV *z* = +4.0 to +5.2; AVC–AB *z* = +4.2 to +5.1). Consistent with this, two Mesp1 clones spanning OFT and RV support shared OFT/RV progenitor states during gastrulation [13].

**TABLE 2:** Robustness of Small‐clone co‐occurrence to the small/large size threshold. Clonal‐coupling z‐scores for all 15 region pairs in the Small‐clone bin, computed at five alternative size thresholds (≤ 20, ≤ 25, ≤ 30, ≤ 35, ≤ 40 cells; columns). Cells with FDR ≤ 0.05 are marked with an asterisk. The ≤ 30 cells column is the threshold used in the main text. Z‐scores and FDR are computed as in Figure 2b and Figure S1. AB–AVC and OFT–RV reach FDR ≤ 0.05 at all five cutoffs; OFT–LV reaches significance only at the widest cutoff (≤ 40 cells).

Region pair	≤20 cells	≤25 cells	≤30 cells	≤35 cells	≤40 cells
OFT‐RV	5.18*	4.42*	4.38*	4.39*	3.97*
AVC‐AB	4.91*	4.96*	4.99*	5.13*	4.20*
OFT‐LV	−1.37	−1.76	−2.09	−2.37	−2.95*
AB‐Atria	1.21	1.33	2.18	1.85	1.72
OFT‐AVC	−1.06	−0.31	−0.55	−1.10	−1.24
OFT‐AB	−1.39	−0.12	−0.47	−0.97	−1.04
OFT‐Atria	−0.49	−0.40	−0.64	−0.64	−0.21
RV‐LV	−1.21	0.14	−0.31	−0.77	−0.78
RV‐AVC	0.36	−0.11	−0.34	−0.90	−0.25
RV‐AB	−1.22	−1.58	−1.79	−2.10	−2.02
RV‐Atria	−0.93	−1.45	−1.64	−1.50	−0.85
LV‐AVC	−0.82	−1.02	−0.29	0.77	0.63
LV‐AB	−1.06	−1.31	−0.92	−0.66	−1.23
LV‐Atria	−1.48	−1.79	−1.55	−0.86	−1.12
AVC‐Atria	−0.04	−0.38	0.11	0.73	1.41

Three small clones were restricted to AVC and AB (mean 12.0 cells), substantially larger than the five AB‐only (5.4 cells) or two AVC‐only (6.0 cells) clones. The nine clones restricted to OFT and RV were similarly larger than their single‐region counterparts (mean 16.1 cells, vs. 6.1 for OFT‐only and 5.0 for RV‐only). Under the size‐as‐time assumption, these larger two‐region clones must therefore have been labelled earlier, when a single progenitor could still give rise to both regions.

A third pair, AB with Atria, showed a positive but nonsignificant trend (*z* = 2.18, raw *p* = 0.029, FDR = 0.14; Figure 2b). The three clones restricted to AB and Atria were also larger than single‐region clones (mean 20.7 cells, vs. 5.4 and 10.9).

## 6 A Potency Graph of Cardiac Fate Restriction

We next asked whether the clonal data are compatible with two separate lineage trees, as in the classical two‐lineage model, or whether they instead support many possible restriction paths. In a strict two‐lineage scenario, from some early point onward there would be two non‐interacting progenitor pools: one contributing OFT but never LV (plus other chambers), and one contributing LV but never OFT (plus other chambers). After this split, no clone would ever contain both OFT and LV descendants [1, 27].

### 6.1 Building a Potency Graph From Clone Patterns

We built a potency graph in three steps:

1. For each clone we recorded the set of cardiac regions it populated (OFT, RV, LV, AVC, AB, Atria). For example, a clone contributing to both LV and Atria would have the pattern {LV, Atria}. Clones with the same set of regions were grouped together. For each distinct pattern we kept (i) the list of regions, (ii) the number of clones showing this pattern, and (iii) the mean clone size.
2. Each distinct pattern became a node. Nodes at the top of the graph correspond to broad, multi‐region potency. Nodes lower down correspond to more restricted regional output.
3. We drew a directed edge from node A to node B if (i) the region set of B is a strict subset of the region set of A, with B having one or two fewer regions compared to A, and (ii) the mean clone size in A is larger than in B (consistent with A representing an earlier, less‐restricted developmental state). An edge from A to B therefore represents a candidate step of regional restriction.

From this potency graph we enumerated all simple paths ending at a terminal. Each path corresponds to a candidate restriction sequence, with internal nodes representing progressively restricted potency states and the terminal node representing a final fate. The graph contains 29 potency states connected by 83 directed edges generating 119 paths (Table S3 and S4). Path lengths range from 1 to 5 restriction steps. The median is 3 and 85% of paths have 3 or 4 steps. Of these 119 paths, 105 begin at the root {OFT, RV, LV, AVC, AB, Atria}.

This analysis does not prove all paths occur in vivo. Instead, it shows which restriction paths are mathematically consistent with the observed clonal patterns, a hypothesis‐generating exercise that maps candidate restriction sequences.

### 6.2 Path‐Implied Descendant Distribution for Each Potency State

We next determined each node's “fate spectrum”, that is, which terminal heart regions can be reached from that node.

We calculated this in two steps:

1. For each potency state, we identified all paths through the graph that pass through it.
2. For each state, we counted how many paths lead to each terminal region (OFT, RV, LV, AVC, AB, or Atria) and converted these counts to proportions (the full per‐state fate spectrum is given in Table S4). For example, if 10 paths pass through a state and 5 end in LV while 5 end in RV, the state has 50% LV and 50% RV in its fate spectrum.

### 6.3 Ordering Potency States

**FIGURE 3:**
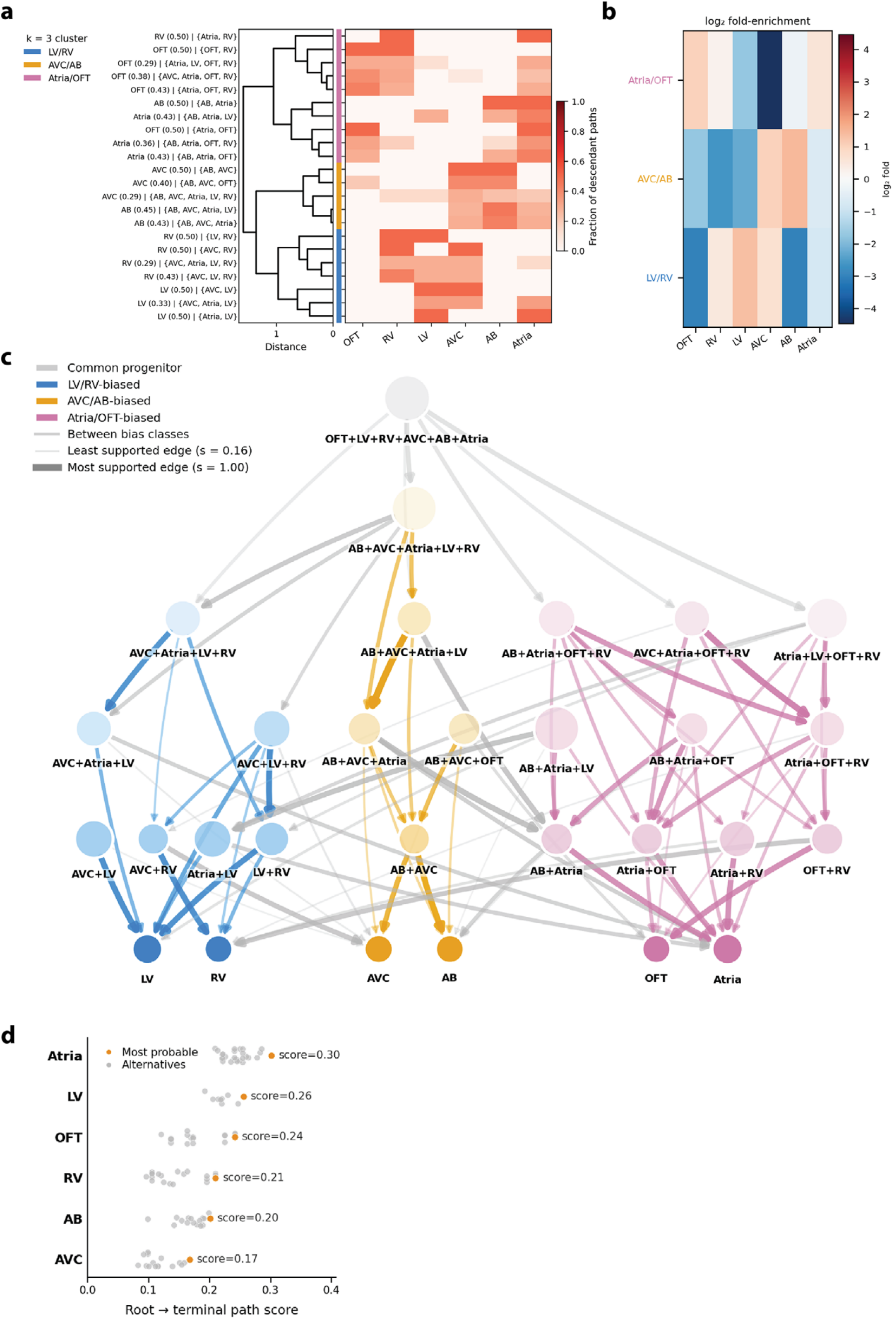
A connected hierarchy of progenitor states with three bias families. (a) Hierarchical clustering of intermediate potency states by their descendant‐fate profiles. Rows are the 22 multi‐region states (single‐region terminals and the 6‐region root excluded). Columns are the six terminal fates. Row labels show each state's dominant fate (with its fraction) and its region set. Cell color gives the fraction of each state's descendant clones terminating in each fate (rows sum to 1). Rows are ordered by Ward‐linkage clustering on the fate‐fraction vectors (Euclidean distance). The *k* = 3 cut (colored strip, left) partitions states into LV/RV‐biased (blue), AVC/AB‐biased (orange), and Atria/OFT‐biased (pink) clusters. (b) Log_2_ fold‐enrichment of each cluster's mean fate composition relative to the mean across all 22 internal states. (c) Lineage‐potency hierarchy colored by the *k* = 3 clusters from panel (a). Each node is a potency state defined by the set of cardiac regions it can give rise to; edges denote restriction steps in which one region is lost. The fully multipotent root sits at the top, terminal fates at the bottom. Node size scales with mean clone size. Edge width and opacity scale with the edge support s(A → B), the fraction of clones compatible with A that are also compatible with the step A → B (see Supporting Information for the compatibility rule). (d) Strip plot of every simple root → terminal path score, grouped by terminal fate (rows). Each path's score is the product of the edge supports along the full path. The highest‐scoring path per fate (the “backbone”) is shown in orange and annotated with its value. See also Figure S2. Alternative paths are grey. Backbones occupy a narrow score range and sit close to the next‐ranked alternatives, indicating that no single restriction sequence dominates. See also Table 3.

Having calculated a fate spectrum for each potency state, we asked whether some states have similar terminal‐output profiles and might therefore constitute broad classes of progenitors with related regional tendencies. To visualize these relationships, we performed hierarchical clustering of the fate spectra and displayed the result as a heat map (Figure 3a).

Three bias classes emerged: OFT/atrial‐enriched, ventricular‐enriched, and AVC/AB‐enriched (Figure 3b). OFT‐ and atrial‐enriched states often cluster together despite representing opposite poles of the heart tube [28]. Ventricular‐enriched states (LV and RV) form a separate cluster. AVC/AB‐enriched spectra form an intermediate cluster between the ventricular and OFT/atrial classes.

We then asked whether the potency graph is organized as separate classes (ventricular‐enriched, AVC/AB‐enriched, OFT/Atria‐enriched) or whether transitions frequently connect across them. Most connections link states within the same class (53/83, ∼64%), while the rest connect different classes (30/83, ∼36%). At the level of restriction paths, about half traverse multiple classes (60/119, ∼50%) (Figure 3c). Multipotent states provide links between the ventricular, AVC/AB, and OFT/atrial classes, consistent with a connected family of restriction histories.

### 6.4 A Probabilistic Network of Restriction Steps

All 119 paths are topologically compatible with the clonal data, but they are not equally well supported by it. We therefore score each restriction step by how strongly the clones support it, and combine these along each route into a path score (Supporting Information). No single route to any fate stands out: the scores are spread across many paths.

For each edge A → B in the potency graph (where B's region set is a subset of A's), the edge support (shown by edge width and opacity in Figures 3c and S2) is the fraction of the clones compatible with state A that are also compatible with continuing to B:

(1) $$\begin{array}{c} \text{edge}\;\text{support}\left(A\to B\right)=\frac{\left(\text{clones}\;\text{compatible}\;\text{with}\;\text{the}\;\text{step}A\to B\right)}{\left(\text{clones}\;\text{compatible}\;\text{with}\;\text{state}A\right)}. \end{array}$$

The score of a complete root‐to‐terminal path is the product of the edge supports along it. For each terminal fate the path with the highest score is the backbone for that fate (Figure 3d). Each backbone, however, is one path among many leading to its terminal, and its score makes up only a small fraction of the total support reaching that terminal:

(2) $$\begin{array}{c} \text{Dominance}\left(\text{terminal}\right)=\frac{\text{score}(\text{backbone})}{\Sigma \text{score}(\text{paths}\;\text{to}\;\text{terminal})} \end{array}$$

The median dominance across the six terminals is 7.6% (range 4.5%–12.8%; Table 3), and the backbone's score is at most 1.056× that of the second‐best path (median 1.02×).

**TABLE 3:** Per‐terminal backbone summary for the six terminal fates. Path scores are products of edge supports along the root → terminal path. An edge support (A → B) is the fraction of clones compatible with state A that are also compatible with the step A → B (see Supporting Information). For each terminal, # paths is the number of distinct simple root → terminal paths ending at that fate. The backbone score is the highest of these path scores, and the runner‐up score the second‐highest. The total mass is the sum of all path scores to the terminal. Dominance is the backbone score divided by the total mass, and margin is the backbone score divided by the runner‐up score. For OFT and RV the top two paths are tied (margin = 1.00×). Raw are ordered by backbone score.

Terminal	# paths	Backbone score	Runner‐up score	Total mass	Dominance	Margin
Atria	27	0.301	0.286	6.63	4.5%	1.056×
LV	9	0.256	0.246	2.00	12.8%	1.038×
OFT	17	0.242	0.242	3.11	7.8%	1.000×
RV	20	0.210	0.210	2.94	7.1%	1.000×
AB	16	0.201	0.199	2.73	7.4%	1.011×
AVC	16	0.167	0.159	1.91	8.8%	1.056×

### 6.5 Compatibility With Binary Lineage Topologies

We next asked whether the clonal data single out one bifurcating tree, as a fixed lineage hierarchy would predict, or many. A complete tree must specify how all six terminals are reached. Combining the paths into complete trees gives a large set of candidates. We score each tree by its joint score (the combined support of the paths that make it) evaluated over the full space of path combinations (Supporting Information).

**FIGURE 4:**
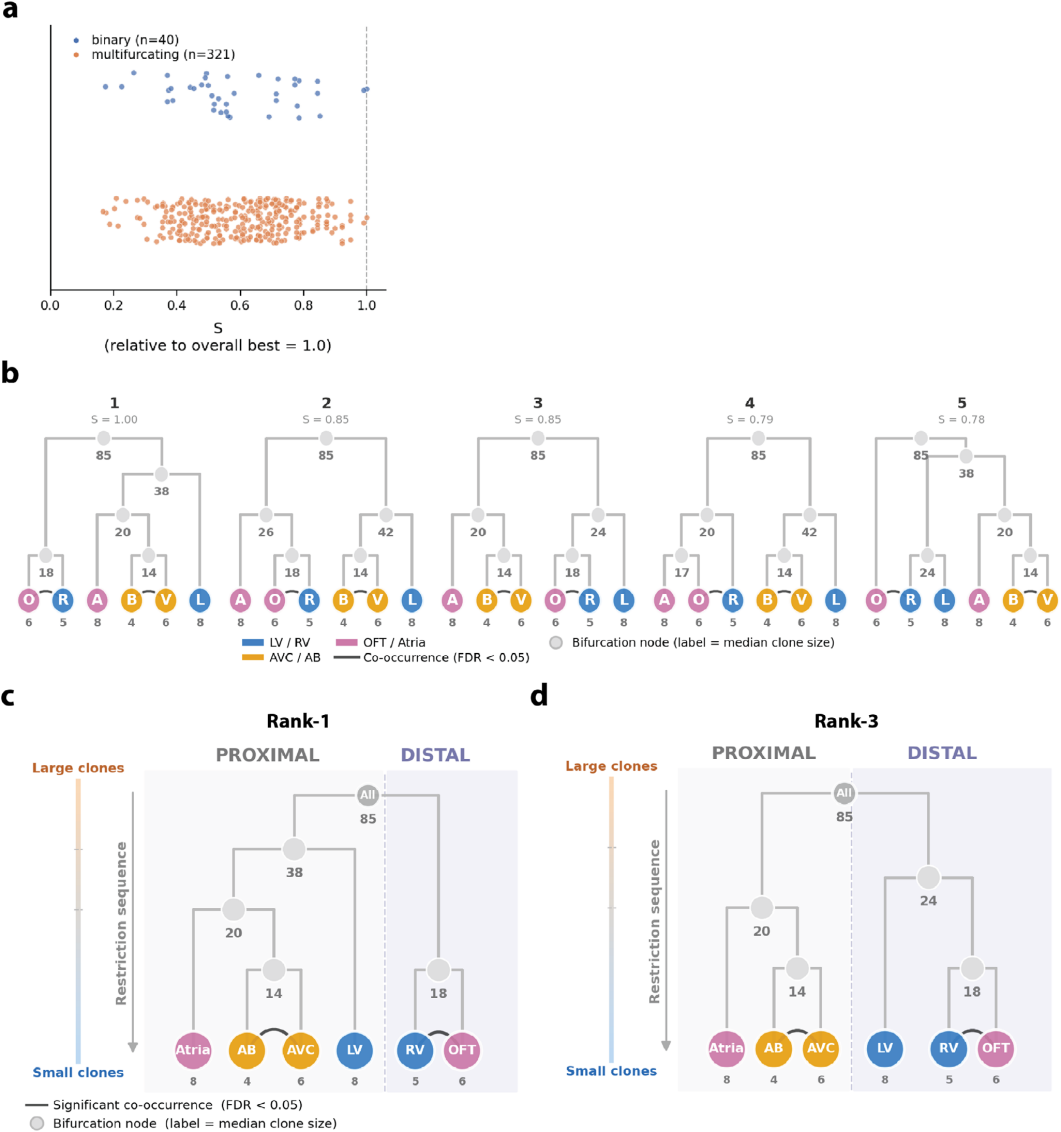
A family of best‐supported restriction histories rather than a single dominant tree. (a) Binary lineage trees fit the clonal data no better than multifurcating ones. Each topology compatible with the data, obtained by exhaustive enumeration of the full space of 21 150 720 root‐to‐terminal path combinations, is plotted by its relative score *S* (= 1.00 at the overall best). Multifurcating topologies allow single states to split into three or more fates at once. The best binary and best multifurcating topologies reach the same *S* (1.00). The full set of 40 binary trees is shown in Figure S3. (b) The five highest‐scoring of the 17 binary topologies surviving the monotone‐median filter (full set in Figure S4; see Supporting Information). Panels are sorted by joint‐score rank, with rank‐1 leftmost; rank‐1 and rank‐3 are shown in detail in (c) and (d). The best scoring binary tree overall fails the monotone‐median rule and is absent here, so the same rank‐1 tree is 0.99 against the whole enumeration used in Figure S3. Root at top, terminals at bottom, coloured by *k* = 3 cluster. Numbers below nodes give the median clone size (cells) of clones supporting that node. Arcs below the terminal row link fate pairs with significant Small‐clone co‐occurrence from Figure 2b (AB–AVC, OFT–RV). (c and d) Rank‐1 and 3 restriction sequences. The vertical gradient bar to the left of the tree relates restriction depth to clone size (large clones at top, small clones at bottom). Background shading separates the proximal from the distal zone. *S* is the relative score (each topology's joint score as a fraction of the top‐ranked topology's; see Supporting Information).

Of the topologies compatible with the data that also pass the permissive size rule (requiring only that at every parent‐child edge the largest clone supporting the parent is strictly larger than the smallest clone supporting the child), 40 are binary and 321 multifurcating, with single states splitting into three or more fates at once (Figures 4a and S3). Most trees compatible with the data are therefore non binary. We acknowledge that the binary constraint is a prior on tree shape, not a finding from the data.

Applying the stricter monotone‐median rule (which keeps a sequence only if the median clone size decreases down the tree) narrows this set to 17 (Figure S4 and Table 4). The five highest‐scoring of the 17 are shown in Figure 4b. 222 multifurcating trees also survive. No single tree dominates: the fifth ranks at 78% as high as the first. No binary tree carries more than 0.12% of the support under either rule (a topology's support weight is the share of the total joint score carried by all path‐combinations that produce that topology; see Supporting Information).

**TABLE 4:** Per‐topology summary for the seventeen surviving topologies, ranked by joint score. The five highest‐scoring are shown in Figure 4b. #: Topology rank (1 = best‐supported). log score. The log of the topology's joint score (less negative values indicate a higher score). Rel. score: The joint score as a percentage of the best topology (rank‐1 = 100%). Rel. score is expressed relative to the best of the 17 surviving topologies, so rank 1= 100%. Support: the summed score of all paths‐combinations producing the topology divided by the summed score over all 21,150,720 combinations. Per‐terminal margin: at each terminal, the ratio of the score of the path this topology uses to the score of the best alternative path to the same terminal; reported as the median across the six terminal. A value of 1.00x means the alternative scores identically. Δ next topo: Ratio of this topology's joint score to that of the next‐ranked topology. Rank 17 has no lower‐ranked topology and is marked “_”. *n* Combos: Number of distinct path combinations yielding the same topology.

#	log score	Rel. score	Support	Per‐terminal margin	Δ next topo	*n* Combos
1	−8.946	100%	0.038%	1.04×	1.17×	4745
2	−9.106	85%	0.064%	1.01×	1.00×	8610
3	−9.107	85%	0.011%	1.06×	1.07×	1596
4	−9.178	79%	0.052%	1.01×	1.02×	8925
5	−9.196	78%	0.017%	1.04×	1.07×	2210
6	−9.266	73%	0.003%	1.04×	1.01×	380
7	−9.276	72%	0.012%	1.01×	1.08×	2646
8	−9.355	66%	0.117%	1.02×	1.17×	20 160
9	−9.515	57%	0.008%	1.03×	1.01×	1470
10	−9.525	56%	0.014%	1.03×	1.00×	3354
11	−9.525	56%	0.010%	1.03×	1.08×	2494
12	−9.600	52%	0.002%	1.02×	1.01×	380
13	−9.610	52%	0.001%	1.01×	1.02×	324
14	−9.631	50%	0.002%	1.03×	1.36×	432
15	−9.935	37%	0.005%	1.02×	1.00×	1404
16	−9.935	37%	0.003%	1.02×	1.63×	1044
17	−10.426	23%	0.001%	1.02×	—	525

Each node has only a few clones, though, so one clone of the right size could in theory be enough to keep a sequence in. We therefore treat the surviving sequences as an illustration of the trees the data permit, not a definitive exclusion of other possibilities. The narrow margins between the surviving sequences may also reflect the limited resolving power of the dataset.

The five sequences differ in where they place the first split (Figure 4b). According to the two‐lineage model, an initial lineage split separates the LV‐containing first lineage from the OFT‐containing second lineage. The third and fifth sequences do not divide this way. For example, the third sequence places OFT, RV and LV on one side and AVC, AB and atria on the other, so OFT and LV stay together rather than separating. The data therefore do not require OFT and LV to split first.

The five sequences share structure (Figure 4b). AVC and AB appear as sister fates in all five, consistent with their strong co‐occurrence. OFT and RV, which also co‐occur significantly, are recovered as sisters in the three highest‐ranked sequences but not in the two lowest. The fourth pairs OFT with Atria, and the fifth separates OFT and RV entirely. The two least‐plausible sequences of the five therefore fail to reproduce the OFT–RV pairing that the co‐occurrence analysis independently supports.

Different progenitors in different embryos may follow different restriction sequences, and less‐supported ones likely occur in the population. A 52‐cell clone spanning RV and Atria, for instance, is incompatible with all 17 surviving binary trees, presumably reflecting a rarer history. Which sequences actually occur in vivo will require live‐imaging.

If fate restriction followed a single deterministic hierarchy, one topology should dominate the joint score, and the best path to each fate should lead its alternatives by a wide margin. Instead, support is spread across a family of comparable topologies, and the data do not single out any one fixed tree.

## 7 Reinterpreting First/Second Lineages With a Connected Landscape

Our analyses recover an LV‐containing/OFT‐excluding sector and an OFT‐containing/LV‐excluding sector, matching the first‐ and second‐lineage clone categories described by Meilhac et al. (Figure 3c) [1]. However, our graph‐based analysis and binary‐tree construction are compatible with a wider range of lineage topologies, and do not single out a two‐tree organization separating OFT and LV.

A two‐lineage model can accommodate individual clonal patterns, but it requires different ad hoc explanations for each. Some clones are labelled before the bifurcation, others after; some are assigned to overlap regions, others to lineage‐specific domains. No single bifurcation point explains all the patterns at once.

A 57‐cell clone contributes to OFT, RV, LV, and Atria but excludes AVC and AB. Under a two‐lineage model [1], a progenitor giving rise to both OFT and LV must sit above the lineage bifurcation. It should therefore also contribute to AVC and AB. This clone does not. A 91‐cell clone contributes to RV, LV, AVC, AB, and Atria but excludes OFT. Under the size‐as‐time assumption from Meilhac et al., a clone this large must derive from an early progenitor sitting above the lineage bifurcation. But such a progenitor should also contribute to OFT, which this clone does not. OFT+LV dual clones (2 of 23 large clones) are no more frequent than OFT+AVC dual clones (2 of 23). This gives no evidence for a unique OFT–LV split underlying the two‐lineage model.

The 105 restriction paths from which the topologies are assembled suggest an alternative picture: a population of progenitors, each contributing to a characteristic set of heart regions, with no single pattern dominating. This is consistent with a probabilistic developmental process, analogous to retinal progenitors, which produce variable clone compositions but stable population‐level ratios [3]. Each large clone would then be a sample from a process in which progenitors share potential but differ in their probability of contributing to each region. In this view, the bias against OFT–LV and OFT–AVC pairings need not reflect a single split into two lineages. It can instead emerge from biased probabilistic fate output. The fact that many distinct lineage trees can fit the Meilhac et al. dataset is consistent with reproducible tissue patterning arising from variable single‐progenitor histories.

## 8 Restriction Sequences and the Spatiotemporal Order of the Primitive Streak

How can the live‐imaging datasets be reconciled with the binary lineage trees we recovered, and can they help discriminate which are more plausible?

Consider founder cells distributed along the proximal–distal axis of the epiblast at gastrulation onset. Their descendants do not disperse in all directions. Lineage tracing in mouse shows that growth in the epiblast is non‐coherent and anisotropically directed toward the primitive streak [29]. Because the spread is directional, the proximal–distal position is preserved as their descendants reach the streak at different times. The first bifurcation in our top‐ranked sequence separates {LV, Atria, AVC, AB} from {OFT, RV} (Rank‐1 in Figures 4d and S2). This split would track the proximal–distal bifurcation. Subsequent bifurcations would then track when each cohort leaves the streak, matching the order of progenitor emergence (Figure 1a). The third sequence instead places LV with the distal OFT and RV branch (Rank‐3 in Figures 4d and S2). Rather than competing with the first sequence, this fits a dual origin for the LV with a proximal Hand1+ contribution and a distal, Foxa2^+^ one [15, 16]. The two sequences need not be mutually exclusive.

Therefore, individual epiblast cells need not be committed to specific cardiac fates. In this view, regional fate is prefigured by the spatial distribution of their descendants in the epiblast.

This mapping is a population‐level tendency. Individual clones generate variable patterns of regionalization. In the large‐clone bin, clone size and regional breadth are independent (Figure 2a). A 42‐cell clone is restricted to Atria, while other 30–40‐cell clones span two to four regions. Founders whose descendants stay clustered reach the streak at one position and contribute to a single region. Those whose descendants spread reach several positions and contribute to several.

Some clones span the proximal–distal boundary, for example {Atria, OFT} at 37 cells and {Atria, OFT, RV} at 32. This fits with founders sitting at the boundary, whose daughters split between the proximal and distal cohorts.

The {OFT, RV} branch matches the Foxa2 lineage‐tracing result, in which outflow‐tract cells arise from progenitors that previously expressed Foxa2 and contributed to the right ventricle [15, 16]. No current live‐imaging or prospective lineage tracing dataset resolves the AVC and AB pairing, so the clonal co‐occurrence stands as a constraint on how future imaging should be read. Testing it will require live reporters for each compartment, with molecular markers to separate AVC from AB.

Live imaging shows fate allocation resolving in the mesoderm. The earlier‐labelled clones from the Meilhac et al. dataset point to biases already in place in the epiblast. Gastrulation links the two. As cells ingress through the primitive streak at different positions and times, they meet distinct signaling environments and enter mesodermal streams biased toward specific cardiac regions [30]. These environments include BMP and Nodal gradients along the proximal–distal axis, and Wnt and FGF signaling. Much of the heart's coarse patterning may therefore be set by where cells are located in the epiblast and when they leave it.

## 9 Implications for Congenital Heart Diseases

Under a deterministic lineage model, a congenital heart defect (CHD) reflects a defect in a specific lineage. A particular progenitor pool fails to develop, and the region it would have populated is malformed. Under a probabilistic model, a CHD may instead reflect a shift in the distribution of fate outcomes across progenitors. Genetic or environmental perturbations could change the probabilities with which epiblast progenitors contribute to different cardiac regions, for example, reducing the probability that any given progenitor contributes to the LV.

Each embryo contains a finite number of progenitors, and each samples independently from this shifted distribution. The number of LV‐fated cells therefore varies between embryos with identical genetic backgrounds and identical perturbations. In some embryos, enough progenitors are still allocated to the LV to build a normal heart. In others, the shift pushes the sampled count below a threshold, and malformation results. This sampling variability could explain the incomplete penetrance that characterizes most CHDs.

## 10 Conclusion

**FIGURE 5:**
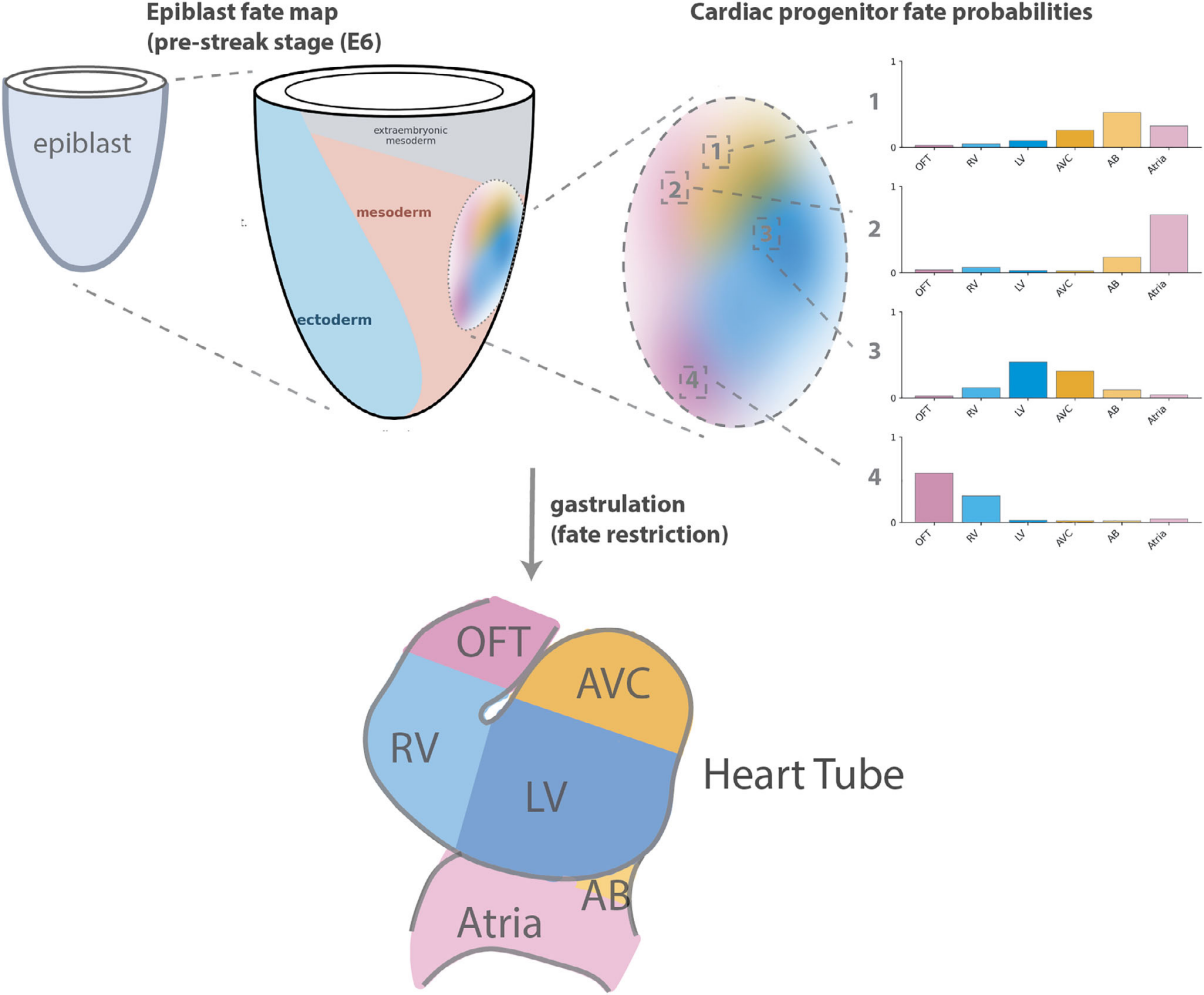
A probabilistic model of cardiac fate restriction. At the pre‐streak stage the epiblast is a cup‐shaped epithelial layer; the multicolored area shows the fate map of the prospective cardiac progenitor region. Enlarged view of the cardiac progenitor region, shaded by the spatial distribution of fate biases. Fate probability distributions for the four positions (1–4), plotted as the probability of contributing to each heart‐tube region. Each progenitor carries a biased but non‐deterministic probability distribution over fates: position 1 favors AB/AVC with some Atria; position 2 favors Atria; position 3 favors LV with some AVC; position 4 favors OFT/RV. During gastrulation, progenitors ingress through the primitive streak and these probability distributions resolve into specific regional allocations in the heart tube at E8.5. LV: left ventricle; RV: right ventricle; OFT: outflow tract, VC: atrioventricular canal; AB: atrial body.

We suggest the question needs reframing. Rather than “which lineage does this progenitor belong to”, the question becomes “what is the probability distribution over fates for progenitors in the epiblast, and how does it shift during gastrulation?” (Figure 5a).

Our probabilistic framing is a working hypothesis that we should test. A quantitative test of the framework would measure clone‐to‐clone variability, as He et al. did for the vertebrate retina [3]. Under a probabilistic model, clones generated from progenitors at similar positions should vary substantially in regional composition while averaging to consistent population‐level proportions. Under a deterministic model, such clones should cluster into a small number of stereotyped composition classes, akin to the patterns Lineage Motif Analysis is designed to detect [31].

Our analysis identifies plausible restriction paths consistent with existing clonal data, but does not establish which specific paths occur in vivo or how frequently. Decisive evidence will come from long‐term live imaging and cell tracking from epiblast to heart tube stages, combined with reporters for different heart compartments. Such experiments, like the time‐lapse studies that tracked every division of individual retinal progenitors in zebrafish, will be needed to link initial cell position, full lineage histories, and terminal fate. These experiments should measure the full clonal distribution, including variance and rare intermediates, and look for gradients rather than sharp boundaries. The aim is not to assign progenitors to one lineage or another but to describe how fate probabilities are distributed across the epiblast and how they change during gastrulation.

## Author Contributions

KI conceived the argument, performed the analysis and wrote the article.

## Conflicts of Interest

The author declares no conflicts of interest.

## Supporting information




**Supporting File**: bies70163‐sup‐0001‐SuppMat.pdf.

## Data Availability

All custom analysis code and processed data are available at https://github.com/Ivanovitch‐Lab/probabilistic‐cardiac‐fate. All randomness uses a fixed seed (0), and a single script (reproduce.sh) regenerates every figure and table from the clone data deposited in the repository. The analysis scripts deposited in the repository were written with the assistance of Anthropic's Claude Code (Claude Opus 4.7, accessed May 2026). The author takes responsibility for the design and correctness of all analyses.
