# Supplementary material for: Beyond Binary: Cardiac Patterning as Probabilistic Fate Restriction
Source: Bioessays. 2026 Aug 19;48(8):e70163. doi: 10.1002/bies.70163 (PMC13488265; doi:10.1002/bies.70163)
Supplement: Supplementary file 1 [file BIES-48-e70163-s001.pdf]

Supplementary Information

Beyond binary: cardiac patterning as probabilistic fate restriction

Kenzo Ivanovitch

Supplementary Figures

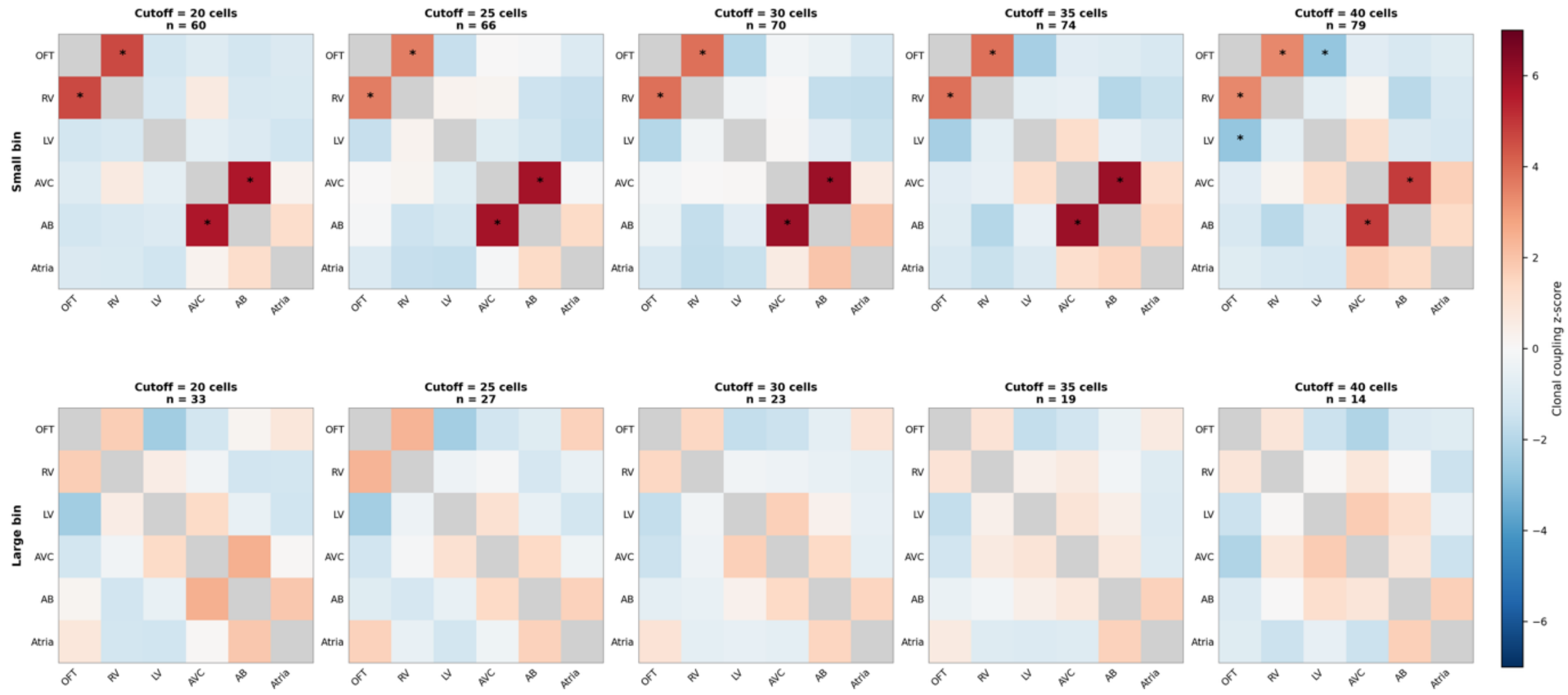

Figure S1. Z-scores for regional co-occurrence computed at five cutoffs (20, 25, 30, 35, 40 cells), separately for Small (≤ cutoff; top row) and Large (> cutoff; bottom row) clones, using the same permutation null model as the main analysis. Warm: positive coupling; cool: negative. Asterisks: FDR ≤ 0.05. Positive OFT–RV and AVC–AB coupling in Small clones is FDR-significant at all cutoffs. In Large clones, OFT–LV negative coupling reaches FDR significance only at cutoff = 20 cells (FDR = 0.018), where the larger sample (n = 33) provides greater power. In Small clones, negative OFT–LV coupling reaches FDR significance only at cutoff = 40 cells (FDR = 0.016).

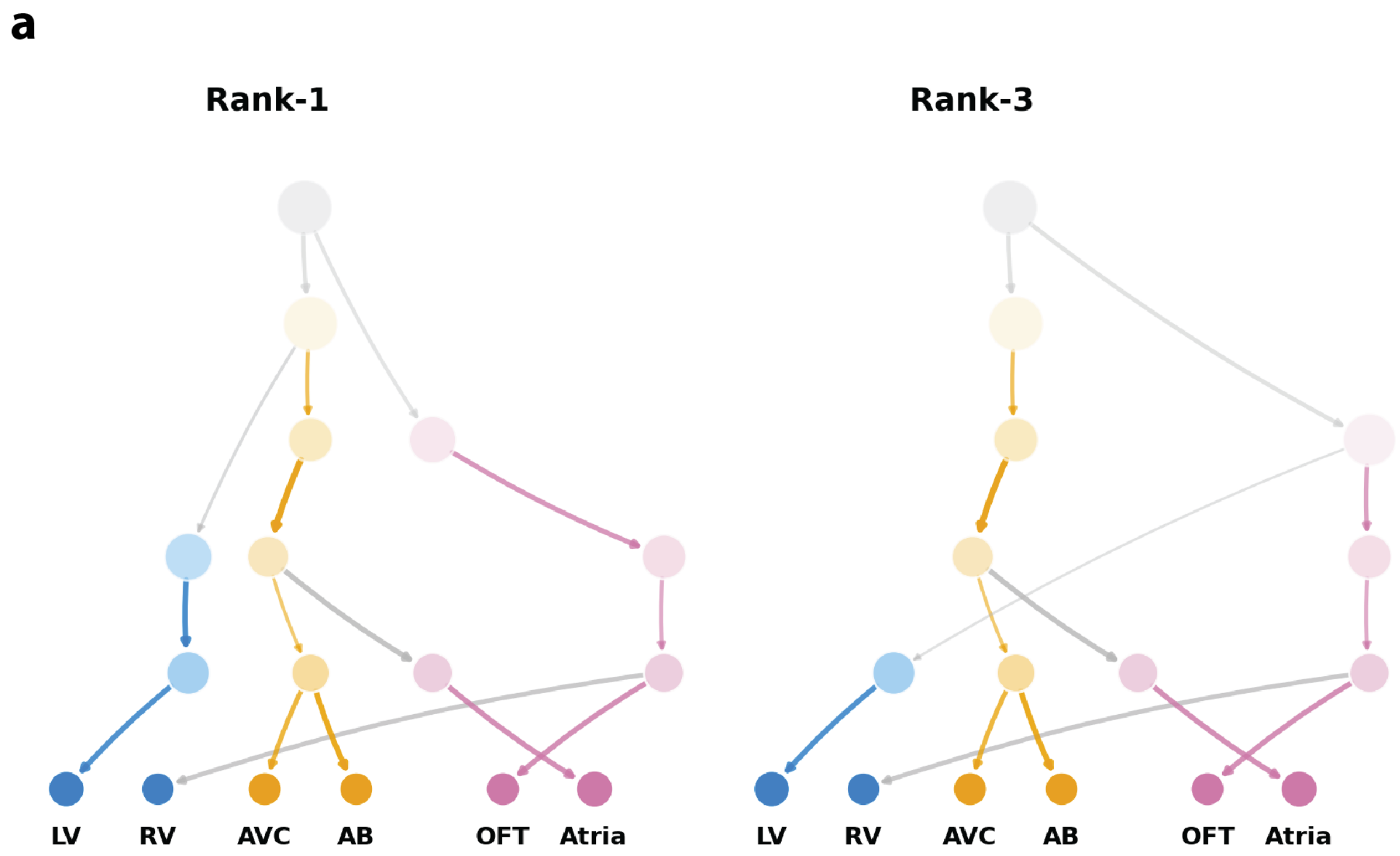

Figure S2. Backbone paths through the potency graph for the rank-1 and rank-3 topologies. Only the backbone nodes and edges of each topology are drawn (rank-1 left, rank-3 right; Figure 4b and Figure 4c). Node colour: k = 3 cluster from Figure 3 (LV/RV blue, AVC/AB orange, OFT/Atria pink). Edge width and opacity: edge support (A → B). The two topologies differ at one branching decision where LV splits off (proximal/junctional pool in rank 1, OFT/RV distal pool in rank 3).

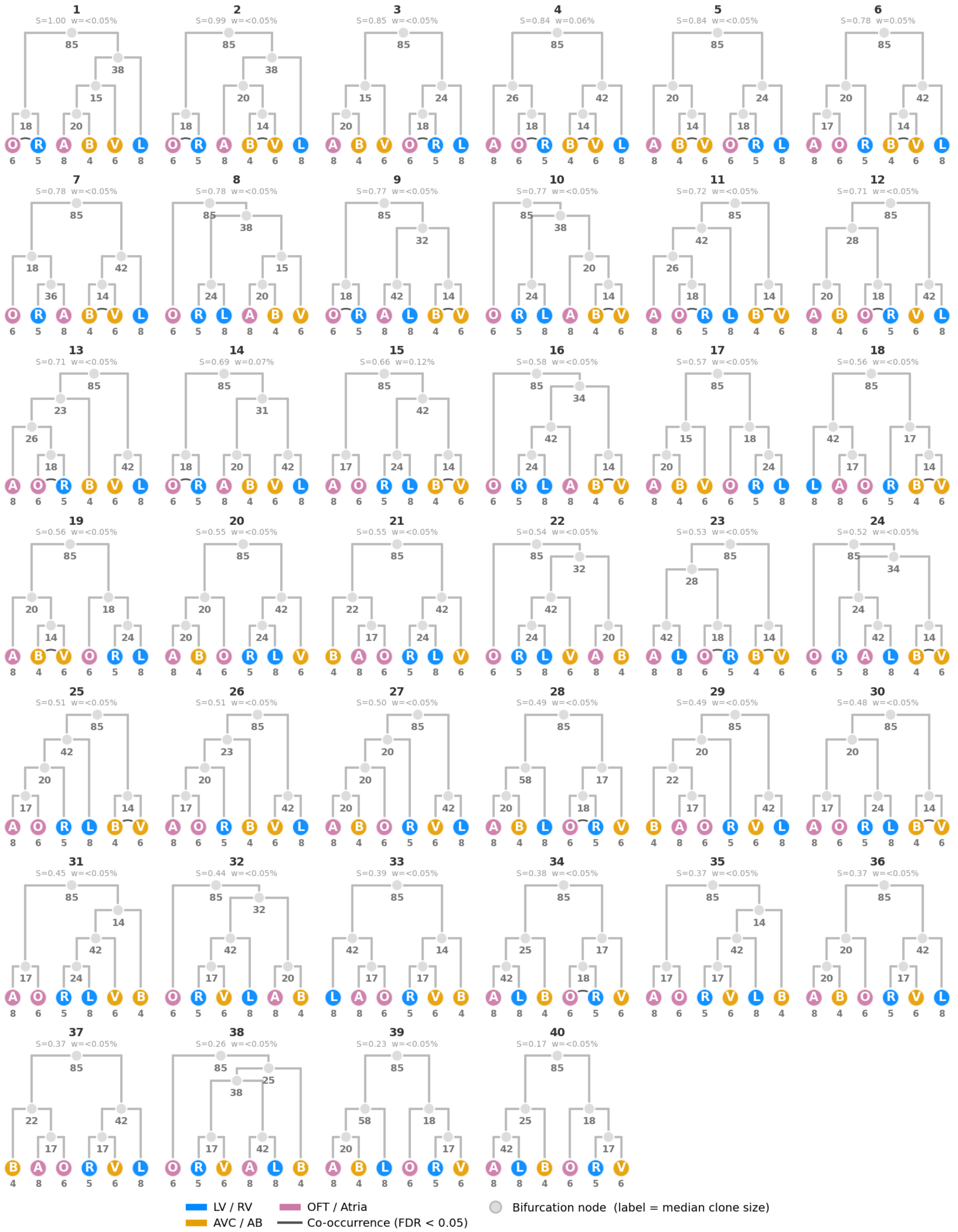

Figure S3. Of the 50 clone-supported strictly-bifurcating topologies recovered from the full enumeration (21,150,720 path combinations), 40 satisfy the weaker size-ordering constraint, requiring only that at every parent–child edge the largest clone supporting the parent is strictly larger than the smallest clone supporting the child (max parent size > min child size). Relative scores S range from 1.00 to 0.17, and no single binary tree carries more than 0.12% of the total support. By comparison, the uniformly best topology under a single-tree model would carry w ≈ 1. The FDR-

significant pairs AB–AVC and OFT–RV are recovered as sisters in 16 and 13 of the 40 trees respectively. S is each topology's best-path score relative to the overall best across all 21,150,720 combinations; w is the joint score summed over every path-combination producing the topology, divided by the same sum across all 21,150,720 combinations (see Supplementary Methods). These 40 trees include the 17 satisfying the stricter monotone-median rule shown in Figure S4.

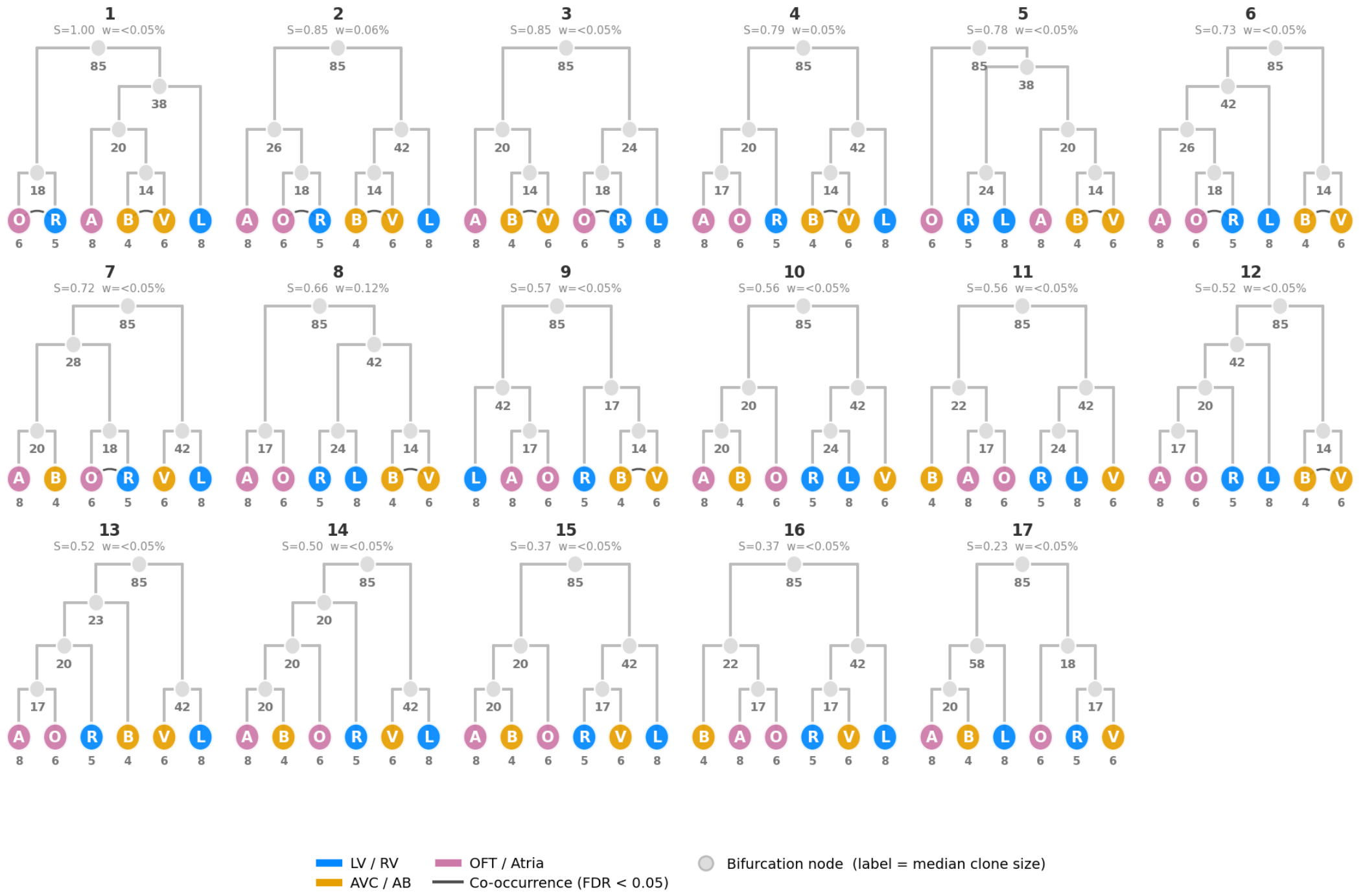

Figure S4. All 17 strictly-bifurcating topologies in which every bifurcation is clone-supported and the median clone size does not increase down the tree (the monotone-median rule), recovered by exhaustive enumeration of the full space of 21,150,720 root-to-terminal path combinations followed by the binary, clone-support and monotone-median filters (see Supplementary Methods). The five highest-scoring are shown in the main text (Figure 4b) and all 17 are tabulated in Table 4. No single tree dominates. S_{17} ranges from 1.00 to 0.23, and no binary tree carries a support weight w above 0.12%. All binary trees together account for 0.88% of the support. The great majority sits on multifurcating topologies (Figure 4a). The FDR-significant pairs AB–AVC and OFT–RV are recovered as sisters in 9 and 5 of the 17 trees respectively, concentrated among the highest-scoring ones (rank-1 and rank-3; Figure 4c,d). Panel labels give S_{17}, each topology's joint score relative to the best of these 17. The best-scoring binary topology overall fails the monotone-median rule and is absent here, so on the whole-enumeration scale used in Figure 4a and Figure S3 the top tree here is 0.99. w is the joint score summed over every path-combination producing the topology, divided by the same sum across all 21,150,720 combinations (see Supplementary Methods).

Supplementary Tables

clone_id	size	bin	R1_OFT	R2_RV	R3_LV	R4_AVC	R5_AB	R6_Atria	n_regions	leaf_regions	leaf_fate_simple
E8.5-171	3	Small	1	0	0	0	0	0	1	OFT	OFT
E8.5-236	5	Small	1	0	0	0	0	0	1	OFT	OFT
E8.5-17	6	Small	1	0	0	0	0	0	1	OFT	OFT
E8.5-199	6	Small	1	0	0	0	0	0	1	OFT	OFT
E8.5-78	6	Small	1	0	0	0	0	0	1	OFT	OFT
E8.5-220	7	Small	1	0	0	0	0	0	1	OFT	OFT
E8.5-10	10	Small	1	0	0	0	0	0	1	OFT	OFT
E8.5-105	3	Small	0	1	0	0	0	0	1	RV	RV
E8.5-209	4	Small	0	1	0	0	0	0	1	RV	RV
E8.5-69	6	Small	0	1	0	0	0	0	1	RV	RV
E8.5-122	7	Small	0	1	0	0	0	0	1	RV	RV
E8.5-124	4	Small	0	0	1	0	0	0	1	LV	LV
E8.5-23	4	Small	0	0	1	0	0	0	1	LV	LV
E8.5-57	4	Small	0	0	1	0	0	0	1	LV	LV
E8.5-208	5	Small	0	0	1	0	0	0	1	LV	LV
E8.5-22	8	Small	0	0	1	0	0	0	1	LV	LV
E8.5-62	8	Small	0	0	1	0	0	0	1	LV	LV
E8.5-89	8	Small	0	0	1	0	0	0	1	LV	LV
E8.5-132	8	Small	0	0	1	0	0	0	1	LV	LV
E8.5-98	11	Small	0	0	1	0	0	0	1	LV	LV
E8.5-159	12	Small	0	0	1	0	0	0	1	LV	LV
E8.5-162	16	Small	0	0	1	0	0	0	1	LV	LV
E8.5-153	27	Small	0	0	1	0	0	0	1	LV	LV
E8.5-180	30	Small	0	0	1	0	0	0	1	LV	LV
E8.5-91	42	Large	0	0	1	0	0	1	2	LV, Atria	LV+Atria
E8.5-106	4	Small	0	0	0	1	0	0	1	AVC	AVC
E8.5-29	8	Small	0	0	0	1	0	0	1	AVC	AVC
E8.5-194	4	Small	0	0	0	0	1	0	1	AB	AB
E8.5-137	4	Small	0	0	0	0	1	0	1	AB	AB
E8.5-32	5	Small	0	0	0	0	1	0	1	AB	AB
E8.5-79	10	Small	0	0	0	0	1	0	1	AB	AB
E8.5-63	4	Small	0	0	0	0	1	0	1	AB	AB
E8.5-178	4	Small	0	0	0	0	0	1	1	Atria	Atria
E8.5-146	4	Small	0	0	0	0	0	1	1	Atria	Atria
E8.5-21	5	Small	0	0	0	0	0	1	1	Atria	Atria
E8.5-224	6	Small	0	0	0	0	0	1	1	Atria	Atria
E8.5-6	7	Small	0	0	0	0	0	1	1	Atria	Atria
E8.5-7	7	Small	0	0	0	0	0	1	1	Atria	Atria
E8.5-116	8	Small	0	0	0	0	0	1	1	Atria	Atria
E8.5-172	8	Small	0	0	0	0	0	1	1	Atria	Atria
E8.5-193	10	Small	0	0	0	0	0	1	1	Atria	Atria
E8.5-20	16	Small	0	0	0	0	0	1	1	Atria	Atria
E8.5-138	16	Small	0	0	0	0	0	1	1	Atria	Atria
E8.5-110	17	Small	0	0	0	0	0	1	1	Atria	Atria
E8.5-221	20	Small	0	0	0	0	0	1	1	Atria	Atria
E8.5-16	25	Small	0	0	0	0	0	1	1	Atria	Atria
E8.5-201	42	Large	0	0	0	0	0	1	1	Atria	Atria
E8.5-43	7	Small	1	1	0	0	0	0	2	OFT, RV	OFT+RV
E8.5-165	11	Small	1	1	0	0	0	0	2	OFT, RV	OFT+RV
E8.5-13	13	Small	1	1	0	0	0	0	2	OFT, RV	OFT+RV
E8.5-145	13	Small	1	1	0	0	0	0	2	OFT, RV	OFT+RV
E8.5-185	18	Small	1	1	0	0	0	0	2	OFT, RV	OFT+RV
E8.5-163	19	Small	1	1	0	0	0	0	2	OFT, RV	OFT+RV
E8.5-184	20	Small	1	1	0	0	0	0	2	OFT, RV	OFT+RV
E8.5-4	20	Small	1	1	0	0	0	0	2	OFT, RV	OFT+RV
E8.5-85	24	Small	1	1	0	0	0	0	2	OFT, RV	OFT+RV
E8.5-128	54	Large	1	1	0	0	0	0	2	OFT, RV	OFT+RV
E8.5-181	21	Small	0	1	1	0	0	0	2	RV, LV	RV+LV
E8.5-175	24	Small	0	1	1	0	0	0	2	RV, LV	RV+LV
E8.5-96	42	Large	0	1	1	0	0	0	2	RV, LV	RV+LV
E8.5-176	42	Large	0	0	1	1	0	0	2	LV, AVC	LV+AVC
E8.5-66	8	Small	0	0	0	1	1	0	2	AVC, AB	AVC+AB
E8.5-135	9	Small	0	0	0	1	1	0	2	AVC, AB	AVC+AB
E8.5-117	19	Small	0	0	0	1	1	0	2	AVC, AB	AVC+AB
E8.5-49	31	Large	0	0	0	1	1	0	2	AVC, AB	AVC+AB
E8.5-15	12	Small	0	0	0	0	1	1	2	AB, Atria	AB+Atria
E8.5-144	20	Small	0	0	0	0	1	1	2	AB, Atria	AB+Atria
E8.5-227	30	Small	0	0	0	0	1	1	2	AB, Atria	AB+Atria
E8.5-177	17	Small	0	1	0	1	0	0	2	RV, AVC	RV+AVC
E8.5-215	41	Large	0	1	1	1	0	0	3	RV, LV, AVC	RV+LV+AVC
E8.5-102	42	Large	0	1	1	1	0	0	3	RV, LV, AVC	RV+LV+AVC
E8.5-68	11	Small	0	0	0	1	1	1	3	AVC, AB, Atria	AVC+AB+Atria
E8.5-75	38	Large	0	0	0	1	1	1	3	AVC, AB, Atria	AVC+AB+Atria
E8.5-218	30	Small	0	0	1	1	1	1	4	LV, AVC, AB, Atria	LV+AVC+AB+Atria
E8.5-72	34	Large	0	0	1	1	1	1	4	LV, AVC, AB, Atria	LV+AVC+AB+Atria
E8.5-77	35	Large	0	0	1	1	0	1	3	LV, AVC, Atria	LV+AVC+Atria
E8.5-139	75	Large	0	0	1	0	1	1	3	LV, AB, Atria	LV+AB+Atria
E8.5-200	22	Small	1	0	0	1	1	0	3	OFT, AVC, AB	OFT+AVC+AB
E8.5-166	36	Large	0	1	1	1	0	1	4	RV, LV, AVC, Atria	RV+LV+AVC+Atria
E8.5-156	48	Large	0	1	1	1	1	1	5	RV, LV, AVC, AB, Atria	RV+LV+AVC+AB+Atria
E8.5-238	52	Large	0	1	0	0	0	1	2	RV, Atria	RV+Atria
E8.5-237	85	Large	0	1	1	1	1	1	5	RV, LV, AVC, AB, Atria	RV+LV+AVC+AB+Atria
E8.5-25	91	Large	0	1	1	1	1	1	5	RV, LV, AVC, AB, Atria	RV+LV+AVC+AB+Atria
E8.5-155	6	Small	1	0	0	0	0	1	2	OFT, Atria	OFT+Atria
E8.5-35	17	Small	1	0	0	0	0	1	2	OFT, Atria	OFT+Atria
E8.5-127	20	Small	0	1	0	0	0	1	2	RV, Atria	RV+Atria
E8.5-60	23	Small	1	0	0	0	1	1	3	OFT, AB, Atria	OFT+AB+Atria
E8.5-14	32	Large	1	1	0	0	0	1	3	OFT, RV, Atria	OFT+RV+Atria
E8.5-191	36	Large	1	1	0	1	0	1	4	OFT, RV, AVC, Atria	OFT+RV+AVC+Atria
E8.5-44	37	Large	1	0	0	0	0	1	2	OFT, Atria	OFT+Atria
E8.5-204	40	Large	1	1	0	0	1	1	4	OFT, RV, AB, Atria	OFT+RV+AB+Atria
E8.5-198	57	Large	1	1	1	0	0	1	4	OFT, RV, LV, Atria	OFT+RV+LV+Atria
E8.5-2	82	Large	1	1	1	1	1	1	6	OFT, RV, LV, AVC, AB, Atria	OFT+RV+LV+AVC+AB+Atria
E8.5-170	142	NaN	1	1	1	1	1	1	6	OFT, RV, LV, AVC, AB, Atria	OFT+RV+LV+AVC+AB+Atria

Table S1. The Meilhac et al. (2004) clones as processed for this paper, one row per clone (n = 94). For each clone we give its regional pattern across OFT, RV, LV, AVC, AB, and Atria, its total laacZ$^+$ cell count, and whether it falls in the small or large bin used throughout the manuscript. Processing of the asterisk-flagged contributions and exclusion of the 142-cell clone, which is retained in the table but left unbinned (bin = NaN) and dropped from downstream analyses, are described in the Supplementary Methods (Input data). See also Box 1.

cutoff	bin	region1	region2	pair	z	p_raw	p_FDR	sig
20	Small	OFT	RV	OFT-RV	5.180	2.21e-07	3.32e-06	True
20	Small	OFT	LV	OFT-LV	-1.371	0.1703	0.4271	False
20	Small	OFT	AVC	OFT-AVC	-1.059	0.2895	0.4357	False
20	Small	OFT	AB	OFT-AB	-1.387	0.1654	0.4271	False
20	Small	OFT	Atria	OFT-Atria	-0.489	0.6247	0.7208	False
20	Small	RV	LV	RV-LV	-1.206	0.2277	0.4271	False
20	Small	RV	AVC	RV-AVC	0.356	0.7216	0.7731	False
20	Small	RV	AB	RV-AB	-1.221	0.2221	0.4271	False
20	Small	RV	Atria	RV-Atria	-0.928	0.3535	0.4821	False
20	Small	LV	AVC	LV-AVC	-0.824	0.4097	0.5121	False
20	Small	LV	AB	LV-AB	-1.057	0.2905	0.4357	False
20	Small	LV	Atria	LV-Atria	-1.476	0.1399	0.4271	False
20	Small	AVC	AB	AVC-AB	4.914	8.90e-07	6.68e-06	True
20	Small	AVC	Atria	AVC-Atria	-0.044	0.9648	0.9648	False
20	Small	AB	Atria	AB-Atria	1.206	0.2278	0.4271	False
20	Large	OFT	RV	OFT-RV	1.177	0.2391	0.5124	False
20	Large	OFT	LV	OFT-LV	-3.235	0.0012	0.0182	True
20	Large	OFT	AVC	OFT-AVC	-1.563	0.1180	0.3441	False
20	Large	OFT	AB	OFT-AB	-0.323	0.7470	0.8432	False
20	Large	OFT	Atria	OFT-Atria	0.331	0.7406	0.8432	False
20	Large	RV	LV	RV-LV	0.806	0.4204	0.7452	False
20	Large	RV	AVC	RV-AVC	0.024	0.9812	0.9812	False
20	Large	RV	AB	RV-AB	-1.485	0.1376	0.3441	False
20	Large	RV	Atria	RV-Atria	-0.597	0.5505	0.8258	False
20	Large	LV	AVC	LV-AVC	1.610	0.1073	0.3441	False
20	Large	LV	AB	LV-AB	-0.513	0.6079	0.8289	False
20	Large	LV	Atria	LV-Atria	-0.760	0.4471	0.7452	False
20	Large	AVC	AB	AVC-AB	2.030	0.0423	0.2342	False
20	Large	AVC	Atria	AVC-Atria	0.270	0.7870	0.8432	False
20	Large	AB	Atria	AB-Atria	1.988	0.0468	0.2342	False
25	Small	OFT	RV	OFT-RV	4.422	9.80e-06	7.35e-05	True
25	Small	OFT	LV	OFT-LV	-1.759	0.0785	0.2945	False
25	Small	OFT	AVC	OFT-AVC	-0.305	0.7601	0.9096	False
25	Small	OFT	AB	OFT-AB	-0.118	0.9064	0.9096	False
25	Small	OFT	Atria	OFT-Atria	-0.395	0.6927	0.9096	False
25	Small	RV	LV	RV-LV	0.145	0.8849	0.9096	False
25	Small	RV	AVC	RV-AVC	-0.114	0.9096	0.9096	False
25	Small	RV	AB	RV-AB	-1.576	0.1151	0.3454	False
25	Small	RV	Atria	RV-Atria	-1.449	0.1472	0.3575	False
25	Small	LV	AVC	LV-AVC	-1.023	0.3065	0.5108	False
25	Small	LV	AB	LV-AB	-1.309	0.1907	0.3575	False
25	Small	LV	Atria	LV-Atria	-1.786	0.0742	0.2945	False
25	Small	AVC	AB	AVC-AB	4.964	6.90e-07	1.03e-05	True
25	Small	AVC	Atria	AVC-Atria	-0.384	0.7013	0.9096	False
25	Small	AB	Atria	AB-Atria	1.327	0.1846	0.3575	False
25	Large	OFT	RV	OFT-RV	1.800	0.0718	0.3066	False
25	Large	OFT	LV	OFT-LV	-2.639	0.0083	0.1249	False
25	Large	OFT	AVC	OFT-AVC	-1.922	0.0546	0.3066	False
25	Large	OFT	AB	OFT-AB	-1.269	0.2045	0.4383	False
25	Large	OFT	Atria	OFT-Atria	0.803	0.4222	0.6333	False

Table S2 (continued on the next page)

cutoff	bin	region1	region2	pair	z	p_raw	p_FDR	sig
25	Large	RV	LV	RV-LV	-0.080	0.9362	0.9802	False
25	Large	RV	AVC	RV-AVC	0.273	0.7847	0.9802	False
25	Large	RV	AB	RV-AB	-0.972	0.3310	0.6206	False
25	Large	RV	Atria	RV-Atria	-0.031	0.9755	0.9802	False
25	Large	LV	AVC	LV-AVC	1.552	0.1207	0.3405	False
25	Large	LV	AB	LV-AB	-0.241	0.8098	0.9802	False
25	Large	LV	Atria	LV-Atria	-0.888	0.3747	0.6245	False
25	Large	AVC	AB	AVC-AB	1.490	0.1362	0.3405	False
25	Large	AVC	Atria	AVC-Atria	-0.025	0.9802	0.9802	False
25	Large	AB	Atria	AB-Atria	1.741	0.0818	0.3066	False
30	Small	OFT	RV	OFT-RV	4.385	1.16e-05	8.71e-05	True
30	Small	OFT	LV	OFT-LV	-2.088	0.0368	0.1381	False
30	Small	OFT	AVC	OFT-AVC	-0.548	0.5835	0.8249	False
30	Small	OFT	AB	OFT-AB	-0.466	0.6409	0.8249	False
30	Small	OFT	Atria	OFT-Atria	-0.641	0.5213	0.8249	False
30	Small	RV	LV	RV-LV	-0.308	0.7580	0.8249	False
30	Small	RV	AVC	RV-AVC	-0.341	0.7333	0.8249	False
30	Small	RV	AB	RV-AB	-1.786	0.0741	0.2224	False
30	Small	RV	Atria	RV-Atria	-1.645	0.1000	0.2501	False
30	Small	LV	AVC	LV-AVC	-0.292	0.7699	0.8249	False
30	Small	LV	AB	LV-AB	-0.918	0.3589	0.6729	False
30	Small	LV	Atria	LV-Atria	-1.551	0.1209	0.2592	False
30	Small	AVC	AB	AVC-AB	4.992	5.98e-07	8.96e-06	True
30	Small	AVC	Atria	AVC-Atria	0.105	0.9160	0.9160	False
30	Small	AB	Atria	AB-Atria	2.181	0.0292	0.1381	False
30	Large	OFT	RV	OFT-RV	1.425	0.1543	0.4628	False
30	Large	OFT	LV	OFT-LV	-2.437	0.0148	0.2220	False
30	Large	OFT	AVC	OFT-AVC	-2.155	0.0312	0.2337	False
30	Large	OFT	AB	OFT-AB	-1.099	0.2718	0.5824	False
30	Large	OFT	Atria	OFT-Atria	0.738	0.4608	0.8443	False
30	Large	RV	LV	RV-LV	0.381	0.7034	0.8443	False
30	Large	RV	AVC	RV-AVC	0.007	0.9943	0.9943	False
30	Large	RV	AB	RV-AB	-0.638	0.5236	0.8443	False
30	Large	RV	Atria	RV-Atria	-0.293	0.7697	0.8443	False
30	Large	LV	AVC	LV-AVC	1.726	0.0844	0.4222	False
30	Large	LV	AB	LV-AB	0.269	0.7880	0.8443	False
30	Large	LV	Atria	LV-Atria	-0.297	0.7661	0.8443	False
30	Large	AVC	AB	AVC-AB	1.456	0.1453	0.4628	False
30	Large	AVC	Atria	AVC-Atria	-0.559	0.5759	0.8443	False
30	Large	AB	Atria	AB-Atria	1.227	0.2199	0.5498	False
35	Small	OFT	RV	OFT-RV	4.389	1.14e-05	8.54e-05	True
35	Small	OFT	LV	OFT-LV	-2.370	0.0178	0.0890	False
35	Small	OFT	AVC	OFT-AVC	-1.103	0.2702	0.5251	False
35	Small	OFT	AB	OFT-AB	-0.969	0.3326	0.5251	False
35	Small	OFT	Atria	OFT-Atria	-0.635	0.5251	0.5251	False
35	Small	RV	LV	RV-LV	-0.767	0.4430	0.5251	False
35	Small	RV	AVC	RV-AVC	-0.899	0.3686	0.5251	False
35	Small	RV	AB	RV-AB	-2.095	0.0361	0.1355	False
35	Small	RV	Atria	RV-Atria	-1.497	0.1344	0.3361	False
35	Small	LV	AVC	LV-AVC	0.775	0.4386	0.5251	False

Table S2 (continued on the next page)

cutoff	bin	region1	region2	pair	z	p_raw	p_FDR	sig
35	Small	LV	AB	LV-AB	-0.664	0.5066	0.5251	False
35	Small	LV	Atria	LV-Atria	-0.863	0.3879	0.5251	False
35	Small	AVC	AB	AVC-AB	5.128	2.92e-07	4.39e-06	True
35	Small	AVC	Atria	AVC-Atria	0.734	0.4629	0.5251	False
35	Small	AB	Atria	AB-Atria	1.851	0.0641	0.1924	False
35	Large	OFT	RV	OFT-RV	0.674	0.5003	0.6919	False
35	Large	OFT	LV	OFT-LV	-2.330	0.0198	0.2974	False
35	Large	OFT	AVC	OFT-AVC	-1.680	0.0930	0.4651	False
35	Large	OFT	AB	OFT-AB	-0.762	0.4459	0.6919	False
35	Large	OFT	Atria	OFT-Atria	0.381	0.7036	0.8118	False
35	Large	RV	LV	RV-LV	0.699	0.4846	0.6919	False
35	Large	RV	AVC	RV-AVC	0.929	0.3529	0.6919	False
35	Large	RV	AB	RV-AB	-0.110	0.9121	0.9121	False
35	Large	RV	Atria	RV-Atria	-0.663	0.5074	0.6919	False
35	Large	LV	AVC	LV-AVC	1.336	0.1816	0.6810	False
35	Large	LV	AB	LV-AB	0.217	0.8282	0.8874	False
35	Large	LV	Atria	LV-Atria	-0.960	0.3372	0.6919	False
35	Large	AVC	AB	AVC-AB	0.852	0.3940	0.6919	False
35	Large	AVC	Atria	AVC-Atria	-0.549	0.5832	0.7290	False
35	Large	AB	Atria	AB-Atria	1.754	0.0795	0.4651	False
40	Small	OFT	RV	OFT-RV	3.970	7.18e-05	5.39e-04	True
40	Small	OFT	LV	OFT-LV	-2.945	0.0032	0.0161	True
40	Small	OFT	AVC	OFT-AVC	-1.239	0.2153	0.4115	False
40	Small	OFT	AB	OFT-AB	-1.038	0.2992	0.4489	False
40	Small	OFT	Atria	OFT-Atria	-0.206	0.8369	0.8369	False
40	Small	RV	LV	RV-LV	-0.781	0.4350	0.5437	False
40	Small	RV	AVC	RV-AVC	-0.253	0.8005	0.8369	False
40	Small	RV	AB	RV-AB	-2.025	0.0429	0.1609	False
40	Small	RV	Atria	RV-Atria	-0.849	0.3961	0.5402	False
40	Small	LV	AVC	LV-AVC	0.633	0.5269	0.6080	False
40	Small	LV	AB	LV-AB	-1.228	0.2195	0.4115	False
40	Small	LV	Atria	LV-Atria	-1.116	0.2643	0.4405	False
40	Small	AVC	AB	AVC-AB	4.199	2.68e-05	4.02e-04	True
40	Small	AVC	Atria	AVC-Atria	1.407	0.1596	0.3989	False
40	Small	AB	Atria	AB-Atria	1.721	0.0853	0.2560	False
40	Large	OFT	RV	OFT-RV	0.805	0.4211	0.6183	False
40	Large	OFT	LV	OFT-LV	-1.268	0.2049	0.6183	False
40	Large	OFT	AVC	OFT-AVC	-1.776	0.0758	0.6183	False
40	Large	OFT	AB	OFT-AB	-1.123	0.2616	0.6183	False
40	Large	OFT	Atria	OFT-Atria	-0.719	0.4722	0.6183	False
40	Large	RV	LV	RV-LV	0.168	0.8663	0.8916	False
40	Large	RV	AVC	RV-AVC	0.806	0.4201	0.6183	False
40	Large	RV	AB	RV-AB	-0.136	0.8916	0.8916	False
40	Large	RV	Atria	RV-Atria	-0.684	0.4938	0.6183	False
40	Large	LV	AVC	LV-AVC	1.701	0.0889	0.6183	False
40	Large	LV	AB	LV-AB	1.058	0.2899	0.6183	False
40	Large	LV	Atria	LV-Atria	-0.181	0.8564	0.8916	False
40	Large	AVC	AB	AVC-AB	0.683	0.4946	0.6183	False
40	Large	AVC	Atria	AVC-Atria	-1.226	0.2201	0.6183	False
40	Large	AB	Atria	AB-Atria	1.481	0.1386	0.6183	False

Table S2. Sensitivity sweep of clonal-coupling z-scores across size-binning cutoffs. One row per (cutoff, bin, region-pair) triple, underlying Figure S1. The z-score computation, null-model construction, and FDR correction are described in Supplementary Methods (Co-occurrence analysis*).*

parent_regions	parent_n	parent_mean_size	child_regions	child_n	child_mean_size	delta_size	regions_lost
AB, AVC, Atria, LV, OFT, RV	1	82.0	AB, AVC, Atria, LV, RV	3	74.7	7.3	1
AB, AVC, Atria, LV, OFT, RV	1	82.0	AB, AVC, Atria, LV	2	32.0	50.0	2
AB, AVC, Atria, LV, OFT, RV	1	82.0	AB, Atria, OFT, RV	1	40.0	42.0	2
AB, AVC, Atria, LV, OFT, RV	1	82.0	AVC, Atria, LV, RV	1	36.0	46.0	2
AB, AVC, Atria, LV, OFT, RV	1	82.0	AVC, Atria, OFT, RV	1	36.0	46.0	2
AB, AVC, Atria, LV, OFT, RV	1	82.0	Atria, LV, OFT, RV	1	57.0	25.0	2
AB, AVC, Atria, LV, RV	3	74.7	AB, AVC, Atria, LV	2	32.0	42.7	1
AB, AVC, Atria, LV, RV	3	74.7	AB, AVC, Atria	2	24.5	50.2	2
AB, AVC, Atria, LV, RV	3	74.7	AVC, Atria, LV, RV	1	36.0	38.7	1
AB, AVC, Atria, LV, RV	3	74.7	AVC, Atria, LV	1	35.0	39.7	2
AB, AVC, Atria, LV, RV	3	74.7	AVC, LV, RV	2	41.5	33.2	2
AB, AVC, Atria, LV	2	32.0	AB, AVC, Atria	2	24.5	7.5	1
AB, AVC, Atria, LV	2	32.0	AB, AVC	4	16.8	15.2	2
AB, AVC, Atria, LV	2	32.0	AB, Atria	3	20.7	11.3	2
AB, AVC, Atria	2	24.5	AB, AVC	4	16.8	7.8	1
AB, AVC, Atria	2	24.5	AB, Atria	3	20.7	3.8	1
AB, AVC, Atria	2	24.5	AB	5	5.4	19.1	2
AB, AVC, Atria	2	24.5	AVC	2	6.0	18.5	2
AB, AVC, Atria	2	24.5	Atria	15	13.0	11.5	2
AB, AVC, OFT	1	22.0	AB, AVC	4	16.8	5.2	1
AB, AVC, OFT	1	22.0	AB	5	5.4	16.6	2
AB, AVC, OFT	1	22.0	AVC	2	6.0	16.0	2
AB, AVC, OFT	1	22.0	OFT	7	6.1	15.9	2
AB, AVC	4	16.8	AB	5	5.4	11.3	1
AB, AVC	4	16.8	AVC	2	6.0	10.8	1
AB, Atria, LV	1	75.0	AB, Atria	3	20.7	54.3	1
AB, Atria, LV	1	75.0	AB	5	5.4	69.6	2
AB, Atria, LV	1	75.0	Atria, LV	1	42.0	33.0	1
AB, Atria, LV	1	75.0	Atria	15	13.0	62.0	2
AB, Atria, LV	1	75.0	LV	13	11.2	63.8	2
AB, Atria, OFT, RV	1	40.0	AB, Atria, OFT	1	23.0	17.0	1
AB, Atria, OFT, RV	1	40.0	AB, Atria	3	20.7	19.3	2
AB, Atria, OFT, RV	1	40.0	Atria, OFT, RV	1	32.0	8.0	1
AB, Atria, OFT, RV	1	40.0	Atria, OFT	3	20.0	20.0	2
AB, Atria, OFT, RV	1	40.0	Atria, RV	2	36.0	4.0	2
AB, Atria, OFT, RV	1	40.0	OFT, RV	10	19.9	20.1	2
AB, Atria, OFT	1	23.0	AB, Atria	3	20.7	2.3	1
AB, Atria, OFT	1	23.0	AB	5	5.4	17.6	2
AB, Atria, OFT	1	23.0	Atria, OFT	3	20.0	3.0	1
AB, Atria, OFT	1	23.0	Atria	15	13.0	10.0	2
AB, Atria, OFT	1	23.0	OFT	7	6.1	16.9	2
AB, Atria	3	20.7	AB	5	5.4	15.3	1
AB, Atria	3	20.7	Atria	15	13.0	7.7	1
AVC, Atria, LV, RV	1	36.0	AVC, Atria, LV	1	35.0	1.0	1
AVC, Atria, LV, RV	1	36.0	AVC, RV	1	17.0	19.0	2
AVC, Atria, LV, RV	1	36.0	LV, RV	3	29.0	7.0	2
AVC, Atria, LV	1	35.0	AVC	2	6.0	29.0	2
AVC, Atria, LV	1	35.0	Atria	15	13.0	22.0	2
AVC, Atria, LV	1	35.0	LV	13	11.2	23.8	2
AVC, Atria, OFT, RV	1	36.0	AVC, RV	1	17.0	19.0	2
AVC, Atria, OFT, RV	1	36.0	Atria, OFT, RV	1	32.0	4.0	1
AVC, Atria, OFT, RV	1	36.0	Atria, OFT	3	20.0	16.0	2
AVC, Atria, OFT, RV	1	36.0	OFT, RV	10	19.9	16.1	2
AVC, LV, RV	2	41.5	AVC, RV	1	17.0	24.5	1
AVC, LV, RV	2	41.5	AVC	2	6.0	35.5	2
AVC, LV, RV	2	41.5	LV, RV	3	29.0	12.5	1
AVC, LV, RV	2	41.5	LV	13	11.2	30.3	2
AVC, LV, RV	2	41.5	RV	4	5.0	36.5	2
AVC, LV	1	42.0	AVC	2	6.0	36.0	1
AVC, LV	1	42.0	LV	13	11.2	30.8	1
AVC, RV	1	17.0	AVC	2	6.0	11.0	1
AVC, RV	1	17.0	RV	4	5.0	12.0	1
Atria, LV, OFT, RV	1	57.0	Atria, LV	1	42.0	15.0	2
Atria, LV, OFT, RV	1	57.0	Atria, OFT, RV	1	32.0	25.0	1
Atria, LV, OFT, RV	1	57.0	Atria, OFT	3	20.0	37.0	2
Atria, LV, OFT, RV	1	57.0	Atria, RV	2	36.0	21.0	2
Atria, LV, OFT, RV	1	57.0	LV, RV	3	29.0	28.0	2
Atria, LV, OFT, RV	1	57.0	OFT, RV	10	19.9	37.1	2
Atria, LV	1	42.0	Atria	15	13.0	29.0	1
Atria, LV	1	42.0	LV	13	11.2	30.8	1
Atria, OFT, RV	1	32.0	Atria, OFT	3	20.0	12.0	1
Atria, OFT, RV	1	32.0	Atria	15	13.0	19.0	2
Atria, OFT, RV	1	32.0	OFT, RV	10	19.9	12.1	1
Atria, OFT, RV	1	32.0	OFT	7	6.1	25.9	2
Atria, OFT, RV	1	32.0	RV	4	5.0	27.0	2
Atria, OFT	3	20.0	Atria	15	13.0	7.0	1
Atria, OFT	3	20.0	OFT	7	6.1	13.9	1
Atria, RV	2	36.0	Atria	15	13.0	23.0	1
Atria, RV	2	36.0	RV	4	5.0	31.0	1
LV, RV	3	29.0	LV	13	11.2	17.8	1
LV, RV	3	29.0	RV	4	5.0	24.0	1
OFT, RV	10	19.9	OFT	7	6.1	13.8	1
OFT, RV	10	19.9	RV	4	5.0	14.9	1

Table S3. Each of the 83 rows is one directed edge of the potency graph (Figure 3). For each edge we give the regional pattern of the parent and the child, the number of clones and the mean clone size for each, and the drop in mean clone size from parent to child (delta_size). A final column (regions_lost) records how many regions are lost across the edge, one or two, since the construction rule allows both. Graph construction is described in the main text and the Supplementary Methods (Potency graph).

node_regions	n_regions	n_clones	mean_size	n_paths_through	dominant_fate	dominant_frac	frac_OFT	frac_RV	frac_LV	frac_AVC	frac_AB	frac_Atria	cluster_k3
AB, AVC, Atria, LV, OFT, RV	6	1	82.0	105	Atria	0.257	0.162	0.190	0.086	0.152	0.152	0.257	—
AB, AVC, Atria, LV, RV	5	3	74.7	35	AVC	0.286	0.000	0.143	0.143	0.286	0.229	0.200	2
AB, AVC, Atria, LV	4	2	32.0	22	AB	0.455	0.000	0.000	0.000	0.273	0.455	0.273	2
AB, AVC, Atria	3	2	24.5	21	AB	0.429	0.000	0.000	0.000	0.286	0.429	0.286	2
AB, AVC	2	4	16.8	12	AVC	0.500	0.000	0.000	0.000	0.500	0.500	0.000	2
AB	1	5	5.4	20	AB	1.000	0.000	0.000	0.000	0.000	1.000	0.000	—
AB, Atria	2	3	20.7	16	AB	0.500	0.000	0.000	0.000	0.000	0.500	0.500	1
AB, Atria, OFT, RV	4	1	40.0	22	Atria	0.364	0.318	0.182	0.000	0.000	0.136	0.364	1
AB, Atria, OFT	3	1	23.0	7	Atria	0.429	0.286	0.000	0.000	0.000	0.286	0.429	1
AVC	1	2	6.0	19	AVC	1.000	0.000	0.000	0.000	1.000	0.000	0.000	—
AVC, Atria, LV, RV	4	1	36.0	14	RV	0.286	0.000	0.286	0.286	0.286	0.000	0.143	3
AVC, Atria, LV	3	1	35.0	9	LV	0.333	0.000	0.000	0.333	0.333	0.000	0.333	3
AVC, RV	2	1	17.0	8	RV	0.500	0.000	0.500	0.000	0.500	0.000	0.000	3
AVC, LV, RV	3	2	41.5	7	RV	0.429	0.000	0.429	0.286	0.286	0.000	0.000	3
AVC, Atria, OFT, RV	4	1	36.0	13	OFT	0.385	0.385	0.308	0.000	0.077	0.000	0.231	1
Atria	1	15	13.0	30	Atria	1.000	0.000	0.000	0.000	0.000	0.000	1.000	—
Atria, OFT	2	3	20.0	14	OFT	0.500	0.500	0.000	0.000	0.000	0.000	0.500	1
Atria, OFT, RV	3	1	32.0	21	OFT	0.429	0.429	0.286	0.000	0.000	0.000	0.286	1
Atria, RV	2	2	36.0	4	RV	0.500	0.000	0.500	0.000	0.000	0.000	0.500	1
Atria, LV, OFT, RV	4	1	57.0	17	OFT	0.294	0.294	0.294	0.118	0.000	0.000	0.294	1
Atria, LV	2	1	42.0	4	LV	0.500	0.000	0.000	0.500	0.000	0.000	0.500	3
LV	1	13	11.2	12	LV	1.000	0.000	0.000	1.000	0.000	0.000	0.000	—
LV, RV	2	3	29.0	8	RV	0.500	0.000	0.500	0.500	0.000	0.000	0.000	3
OFT	1	7	6.1	18	OFT	1.000	1.000	0.000	0.000	0.000	0.000	0.000	—
OFT, RV	2	10	19.9	12	OFT	0.500	0.500	0.500	0.000	0.000	0.000	0.000	1
RV	1	4	5.0	20	RV	1.000	0.000	1.000	0.000	0.000	0.000	0.000	—
AB, AVC, OFT	3	1	22.0	5	AVC	0.400	0.200	0.000	0.000	0.400	0.400	0.000	2
AB, Atria, LV	3	1	75.0	7	Atria	0.429	0.000	0.000	0.286	0.000	0.286	0.429	1
AVC, LV	2	1	42.0	2	LV	0.500	0.000	0.000	0.500	0.500	0.000	0.000	3

Table S4. Per-state summary of the 29 potency states, one row per state. For each state we give its regional pattern, the number of clones and their mean size, the number of root-to-terminal paths passing through it, its fate spectrum (the proportion

of root-to-terminal paths through the state that end in each of the six terminal regions), the most common fate with its proportion (dominant_fate and dominant_frac), and the k = 3 Ward-clustering label (cluster_k3). Clustering uses only the 22 multi-region intermediate states, excluding the single-region terminals and the multipotent root. The procedure is described in the Supplementary Methods (Fate spectrum and clustering). Under the convention used in Figure 3a, cluster 1 is Atria/OFT-biased, cluster 2 is AVC/AB-biased, and cluster 3 is LV/RV-biased.

Supplementary Methods

Input data

The starting point is the published clonal table from Meilhac et al. [1], processed into a binary clone-by-region matrix in which rows are clones (n = 94), columns are the six cardiac regions (outflow tract (OFT), right and left ventricles (RV, LV), atrioventricular canal (AVC), base of the atria (AB) and atria (Atria)), and entries are 1 if the clone contributed to the region and 0 otherwise. Some entries (1–2 cell contributions to a region) are marked with asterisks (*) in the original Meilhac dataset to indicate that clonal relatedness to the main clone cannot be definitively established. Following the original approach [1], we excluded these contributions from co-occurrence analysis by setting them to zero in the binary matrix. The rationale is that for these sparse contributions, there is no definitive way to establish clonal relatedness to the main part of the clone (see Box 1).

The regional pattern of a clone is the set of regions for which the entry is 1; clone size is the total number of laacZ$^+$ cells reported in the original table. The 142-cell clone, which contributes to all six regions, is excluded from the co-occurrence and graph-construction analyses, leaving 93 clones.

Co-occurrence analysis

To test whether certain regions were preferentially co-labelled within clones, we computed a clonal-coupling z-score for each region pair, comparing observed co-occurrences to a degree-preserving null distribution. Positive z-scores indicate co-occurrence above chance. Negative z-scores indicate mutual exclusion.

Within each size bin, we asked: do certain region pairs appear together (or apart) more often than expected by chance? To answer this, we computed a clonal coupling z-score for each region pair. First, we created a null model by randomly shuffling the data 100,000 times while preserving both the number of regions per clone (row sums) and the number of clones per region (column sums). Second, we compared the observed co-occurrence counts to this null distribution. For each region pair, we calculated how many standard deviations the observed count deviated from the random expectation. Positive z-scores indicate regions that co-occur more than expected; negative z-scores indicate mutual exclusion. Third, we determined statistical significance by converting z-scores to p-values and applying false discovery rate (FDR) correction to account for multiple comparisons.

To assess robustness to the size-bin boundary, the full permutation null was repeated at four additional cutoffs (20, 25, 35 and 40 cells) alongside the main cutoff of 30 cells. Clones were reassigned to Small (≤ cutoff) and Large (> cutoff) bins accordingly, and z-scores and FDR-corrected p-values were recomputed independently within each bin using identical settings.

Potency graph

The potency graph is constructed as described in main text, yielding 29 nodes and 83 directed edges. A source state is one with no incoming edge: no observed pattern qualifies as its parent, either because none contains it or because those that do have clones no larger than its own. We enumerated all simple paths from a source state to a terminal, yielding 119 paths. Of these, 105 begin at the fully multipotent root {OFT, RV, LV, AVC, AB, Atria}. The remaining 14 begin at {AB, AVC, OFT}, {AB, Atria, LV} and {AVC, LV}: each is contained in larger observed patterns, but its clones are not smaller, so the size criterion admits no parent.

Fate spectrum and clustering

The fate-spectrum vectors for the 22 multi-region intermediate states (excluding the six single-region terminals and the fully multipotent root) were clustered by hierarchical agglomerative clustering with Ward linkage on Euclidean distances. For the within-class connectivity analysis, class membership uses k = 3 clusters.

Clone-to-path assignment and edge weighting

Each clone's regional pattern corresponds to one node in the graph. Because a clone's pattern usually does not pin down a single restriction route, we treat each clone as evidence on every route compatible with its pattern. It is counted at each state, and along each step, of any root-to-terminal route that passes through its node. Because the graph's nodes are exactly the regional patterns observed in the clones, every clone matches a node and is placed.

The support for a step from a state A to a state B is then the fraction of the clones compatible with A that are also compatible with the step to B:

edge support = (clones compatible with the step A → B) / (clones compatible with state A).

A clone whose pattern is still compatible with several of A's next steps is counted toward each of them, so the supports leaving one state can sum to more than 1. For example, take the three-region state {OFT, RV, LV}, from which one step drops LV (to {OFT, RV}) and another drops OFT (to {RV, LV}). Suppose six clones are compatible with {OFT, RV, LV}: two have exactly that three-region pattern and are therefore compatible with either step, three are more restricted on the {OFT, RV} side, and one on the {RV, LV} side. The step dropping LV is then supported by 2 + 3 = 5 of the 6 clones (0.83) and the step dropping OFT by 2 + 1 = 3 of the 6 (0.50). The two three-region clones are counted toward both steps, which is why the supports sum to more than 1. The support measures how strongly the data back each individual step, not how a fixed pool of cells is divided among siblings.

To score a complete route from the root to a terminal, we multiply the supports of its edges. A route with edge supports 0.50, 0.80 and 0.90 scores 0.50 × 0.80 × 0.90 = 0.36. For each terminal fate, the highest-scoring route is its backbone. Because several routes usually reach the same terminal, we also report each backbone's dominance, i.e. its share of the combined score of all routes to that terminal:

dominance = backbone score / (summed score of all routes to that terminal).

If three routes reach OFT with scores 0.36, 0.30 and 0.28, the backbone (0.36) has a dominance of 0.36 / (0.36 + 0.30 + 0.28) ≈ 0.38, or 38%. A dominance near 1 would mean a single route dominates a terminal; the low values we observe (median 7.6%) instead indicate that the score is spread across many routes, none dominant. For OFT and RV the top two routes score identically, so we pick one and report the tie as margin = 1.000.

Topology enumeration

Each of the six terminals is the endpoint of a set of candidate restriction paths from the root: AB 16, AVC 16, Atria 27, LV 9, OFT 17, and RV 20 paths, the 105 root-originating paths of the 119, and the # paths column of Table 3. A combination chooses one path per terminal. The number of combinations is the product of these counts, 16 × 16 × 27 × 9 × 17 × 20 = 21,150,720. Each combination is scored by the product of its six path scores (or equivalently by the sum of their logarithms).

Many combinations share the same branching structure. We represented each by a canonical signature: the set of its split events, each recorded as the set of fates present above the split together with the sets of fates in the resulting branches. Because the signature is a set of sets, it is independent of the order in which paths were combined, so topologically identical combinations collapse to the same signature. Grouping by signature reduced the 21,150,720 combinations to 639 distinct topologies, each represented by its highest-scoring combination.

Three filters reduce the 639 topologies. 1) The binary filter keeps only topologies in which every internal node has exactly two children: 92 of the 639 are strictly bifurcating, while the remaining 547 contain at least one node that splits three or more ways. 2) Because trees are assembled by combining paths, the merge points can imply ancestral groupings that no observed clone occupies. Such a node ends up empty, and the split it represents is unsupported by the data. Discarding topologies that contain such a node leaves 50 binary and 350 multifurcating. 3) A size-ordering filter is then applied, in two variants. The weaker constraint requires only that at every parent–child edge the largest clone supporting the parent is strictly larger than the smallest clone supporting the child (max parent size > min child size); this leaves 40 binary and 321 multifurcating topologies (Figure 4a, Figure S3). The stricter monotone-median filter keeps only topologies in which each node's median clone size is at least as large as each of its children's, since under the size-as-time assumption an ancestor should not be smaller than its restricted descendants; this leaves 17 binary and 222 multifurcating (Figure S4, Table 4). No single binary tree carries more than 0.12% of the support under either rule (the heaviest multifurcating topology reaches 2.4%).

Each topology was scored by the joint score of its highest-scoring path-combination, the product of its six path scores. Topologies were ranked by this score. Table 4

reports its natural logarithm in the log score column, so that differences between rows are log ratios. Less negative values indicate higher-scoring topologies.

The relative score S is a topology's joint score divided by the joint score of the best topology in the whole enumeration, so the single best-scoring topology has S = 1.00 and every other value is a fraction of it. This is the scale used in Figure 4 and Figure S3. Because the best-scoring binary topology does not survive the monotone-median filter, the 17 surviving topologies are additionally reported on their own scale, S_{17}, relative to the best of that set; this is the Rel. score column of Table 4 and the panel labels of Figure S4. The two scales differ only by a constant factor (the rank-1 tree of Table 4 is S_{17} = 1.00 and S = 0.99).

The support weight w (Support in Table 4) is the summed score of all path-combinations producing a topology, divided by the summed score over all 21,150,720 combinations. Because every combination collapses to exactly one topology signature, the 639 topologies partition the space and their weights sum to 1; a value of w ≈ 1 would mean a single tree accounts for essentially all the score mass, whereas the largest value observed for any binary topology is 0.12%.

The n combos column of Table 4 gives the number of distinct path-combinations producing each topology.

Δ next topo is the ratio of a topology's joint score to that of the topology ranked immediately below it within the same table; a value near 1.00× means the two are nearly indistinguishable. The lowest-ranked row has no topology below it and is marked "—".

The per-terminal margin is computed at each terminal as the ratio of the score of the path this topology uses to the score of the best alternative path to the same terminal, and reported as the median across the six terminals. For OFT and RV the top two paths are exactly tied (Table 3).

Reference:

1. Meilhac SM, Esner M, Kelly RG, Nicolas JF, Buckingham ME. The clonal origin of myocardial cells in different regions of the embryonic mouse heart. Dev Cell. 2004;6(5):685–98. doi: 10.1016/s1534-5807(04)00133-9. PubMed PMID: 15130493.
